# Linkages Among Dissolved Organic Matter Export, Dissolved Metabolites, and Associated Microbial Community Structure Response in the Northwestern Sargasso Sea on a Seasonal Scale

**DOI:** 10.3389/fmicb.2022.833252

**Published:** 2022-03-08

**Authors:** Shuting Liu, Krista Longnecker, Elizabeth B. Kujawinski, Kevin Vergin, Luis M. Bolaños, Stephen J. Giovannoni, Rachel Parsons, Keri Opalk, Elisa Halewood, Dennis A. Hansell, Rod Johnson, Ruth Curry, Craig A. Carlson

**Affiliations:** ^1^Department of Ecology, Evolution and Marine Biology, Marine Science Institute, University of California, Santa Barbara, Santa Barbara, CA, United States; ^2^Department of Marine Chemistry and Geochemistry, Woods Hole Oceanographic Institution, Woods Hole, MA, United States; ^3^Microbial DNA Analytics, Phoenix, OR, United States; ^4^School of Biosciences, University of Exeter, Exeter, United Kingdom; ^5^Department of Microbiology, Oregon State University, Corvallis, OR, United States; ^6^Bermuda Institute of Ocean Sciences, Saint George’s, Bermuda; ^7^Rosenstiel School of Marine and Atmospheric Science, University of Miami, Miami, FL, United States

**Keywords:** dissolved organic matter, amino acids, metabolites, bacterioplankton, Sargasso Sea, seasonal, mixing

## Abstract

Deep convective mixing of dissolved and suspended organic matter from the surface to depth can represent an important export pathway of the biological carbon pump. The seasonally oligotrophic Sargasso Sea experiences annual winter convective mixing to as deep as 300 m, providing a unique model system to examine dissolved organic matter (DOM) export and its subsequent compositional transformation by microbial oxidation. We analyzed biogeochemical and microbial parameters collected from the northwestern Sargasso Sea, including bulk dissolved organic carbon (DOC), total dissolved amino acids (TDAA), dissolved metabolites, bacterial abundance and production, and bacterial community structure, to assess the fate and compositional transformation of DOM by microbes on a seasonal time-scale in 2016–2017. DOM dynamics at the Bermuda Atlantic Time-series Study site followed a general annual trend of DOC accumulation in the surface during stratified periods followed by downward flux during winter convective mixing. Changes in the amino acid concentrations and compositions provide useful indices of diagenetic alteration of DOM. TDAA concentrations and degradation indices increased in the mesopelagic zone during mixing, indicating the export of a relatively less diagenetically altered (i.e., more labile) DOM. During periods of deep mixing, a unique subset of dissolved metabolites, such as amino acids, vitamins, and benzoic acids, was produced or lost. DOM export and compositional change were accompanied by mesopelagic bacterial growth and response of specific bacterial lineages in the SAR11, SAR202, and SAR86 clades, *Acidimicrobiales*, and *Flavobacteria*, during and shortly following deep mixing. Complementary DOM biogeochemistry and microbial measurements revealed seasonal changes in DOM composition and diagenetic state, highlighting microbial alteration of the quantity and quality of DOM in the ocean.

## Introduction

Marine dissolved organic matter (DOM) is produced by various food-web processes such as extracellular release from phytoplankton, zooplankton excretion, grazer sloppy feeding, bacterial release, and viral lysis (Carlson and Hansell, 2015; Koch et al., 2020). Removal of DOM occurs mainly through microbial oxidation by heterotrophic prokaryotes. Therefore, DOM is an important link connecting diverse biological functions in oceanic ecosystems. Its bioavailability ranges from labile DOM that is rapidly consumed to recalcitrant DOM that resists rapid degradation (Carlson and Ducklow, 1995; Carlson, 2002; Hansell, 2013). For instance, a portion of freshly produced dissolved organic carbon (DOC) from phytoplankton was drawn down rapidly within 1–2 weeks in the Southern Ocean and Arctic Ocean, selecting for a distinct bacterial community structure (Landa et al., 2018; Dadaglio et al., 2019); in contrast, a majority of DOC in the water column is composed of compounds of varying degrees of recalcitrance that turn over on a time scale of months to millennia (Carlson and Ducklow, 1995; Hansell, 2013; Carlson and Hansell, 2015).

Microbial oxidation leads to compositional changes of DOM that can be assessed by examining changes in specific components, such as dissolved combined neutral sugars (DCNS) (Skoog and Benner, 1997; Goldberg et al., 2009) or total dissolved amino acids (TDAA). TDAA comprises dissolved free amino acids (DFAA) and amino acids hydrolyzed from proteins, peptides, and other amino acid-embedded polymers (dissolved combined amino acids, DCAA) (Lee and Bada, 1977; Coffin, 1989). TDAA accounts for 0.4–7% of DOC and a larger portion (1.4–15%) of dissolved organic nitrogen (DON) in the ocean (Dittmar et al., 2001; Aluwihare and Meador, 2008; Kaiser and Benner, 2009; Repeta, 2015). Amino acids are one of the most labile components in the DOM pool because they are preferentially degraded by microbes; therefore, their concentrations and compositions can be used as indicators of DOM diagenesis (Suttle et al., 1991; Cowie and Hedges, 1994; Amon et al., 2001). To that end, a commonly used degradation index (DI) is calculated with each amino acid’s relative abundance and its empirical factor coefficient; this index is sensitive to DOM degradation over time scales of weeks to decades (Dauwe et al., 1999; Davis et al., 2009; Kaiser and Benner, 2009).

Metabolomics measures small labile organic molecules that are end products of cellular metabolisms (Fiehn, 2002), and is often analytical challenging (Kido Soule et al., 2015; Boysen et al., 2018). Advanced technologies such as ultrahigh resolution mass spectrometry enable detection of DOM composition at the molecular or compound level, in addition to specific biomolecules such as TDAA (Sleighter and Hatcher, 2008; Lechtenfeld et al., 2014; Kido Soule et al., 2015; Medeiros et al., 2015; Osterholz et al., 2016). Using these techniques, metabolomics, including both particulate and dissolved metabolomics, has primarily been applied to marine culture studies (Baran et al., 2010; Fiore et al., 2015; Longnecker et al., 2015; Kashfi et al., 2020), with more limited applications to field samples (Durham et al., 2019; Johnson et al., 2020; Boysen et al., 2021; Heal et al., 2021). However, seasonal changes of dissolved metabolites in the marine environment are rarely studied.

DOM compositional changes often affect bacterial growth, metabolism, and community structure (Harvey et al., 2006; Båmstedt and Wikner, 2016; Dadaglio et al., 2019). DOM polymers must first be hydrolyzed extracellularly by hydrolytic enzymes to low molecular weight (< 600 Da) prior to uptake by bacterioplankton (Chróst, 1991; Baltar et al., 2010; Arnosti, 2011). Due to different nutrition strategies and hydrolytic efficiencies among different bacteria (Polz et al., 2006; Yooseph et al., 2010; Liu and Liu, 2020; Reintjes et al., 2020), DOM of varying quality may trigger the growth of specific bacterial populations (Covert and Moran, 2001; Alonso-Sáez et al., 2009; Quigley et al., 2019). For instance, fresh DOM input from a phytoplankton bloom, enriched with polysaccharides and TDAA, led to rapid bacterial growth, high bacterial production, and selected copiotrophs such as *Flavobacteria*, *Roseobacter*, and *Pseudoalteromonas* (Buchan et al., 2014; Landa et al., 2016; Balmonte et al., 2019; Avcı et al., 2020). In contrast, recalcitrant DOM resulted in relatively steady and slow bacterial growth of some oligotrophs, such as SAR11, OCS116, and the SAR202 clade that harbors diverse oxidative enzymes involved in recalcitrant DOM degradation (Carlson et al., 2009; Treusch et al., 2009; Landry et al., 2017; Liu et al., 2020a; Saw et al., 2020). However, the bacterioplankton responders can in turn transform labile DOM to a more recalcitrant composition *via* the microbial carbon pump (Amon et al., 2001; Jiao et al., 2010; Romera-Castillo et al., 2011; Goto et al., 2017). Therefore, combining analysis of DOM biogeochemistry and microbial measurements is an important approach to understanding sources and sinks of DOM and mechanisms driving the chemical distribution and biological processes in the ocean.

Deep convective mixing of dissolved and suspended organic matter from the surface to depth represents an important export pathway of the biological carbon pump if mixing is deep enough (Siegel et al., 2016). The northwestern Sargasso Sea experiences annual winter convective mixing, typically to depths of 200–300 m, which exports DOM and suspended particulate organic matter out of the euphotic zone into the upper mesopelagic zone and leads to temporal patterns in DOM and microbial composition in the mesopelagic ocean (Carlson et al., 1994; Hansell and Carlson, 2001; Goldberg et al., 2009; Treusch et al., 2009). The seasonality of the oligotrophic Sargasso Sea provides a unique model system to examine DOM export and subsequent compositional transformation of DOM by microbial oxidation. In this study, we consolidated the first seasonal dataset of TDAA and dissolved metabolites in the northwestern Sargasso Sea to assess the fate and microbial transformation of DOM on a seasonal scale between 2016 and 2017. We also combined DOM biogeochemical dynamics, including bulk DOC, TDAA, and dissolved metabolite concentrations, with corresponding changes in bacterioplankton abundance (BA), bacterial production (BP), and bacterial community structure to examine the roles of specific bacterial lineages in DOM cycling at fine phylogenetic resolution.

## Materials and Methods

### Study Sites and Seawater Collection

Seawater was collected from the northwestern Sargasso Sea located in the North Atlantic subtropical gyre. Samples were collected monthly from July 2016 to September 2017 during regular cruises to the Bermuda Atlantic Time-series Study (BATS) station (31°40′N, 64°10′W). Additional samples were collected during four seasonal process cruises (July 2016, September 2016, April 2017, and July 2017) conducted in the vicinity of BATS or Hydrostation S (HS, 32°10′N, 64°30′W) as part of the Bermuda Institute of Ocean Sciences’ Simons Collaboration on Ocean Processes and Ecology (BIOS-SCOPE) microbial oceanographic program and were combined with the BATS data for more comprehensive analyses of microbial and DOM dynamics of the northwestern Sargasso Sea.

Variables including temperature, salinity, chlorophyll fluorescence, and dissolved oxygen concentrations were measured by sensors attached to the conductivity, temperature, and depth (CTD) profiler. Seawater was collected from 6 to 10 depths within the upper 500 m by 12L Niskin -type sampling bottles (Ocean Test Equipment Inc.) fixed to the CTD rosette. Samples included DOC, TDAA, dissolved metabolites, BA, BP measured by ^3^H-leucine incorporation, and 16S rRNA amplicon sequencing (Table 1). The detailed sampling frequency for each parameter is listed in Table 1. During BATS cruises, one depth profile for targeted parameters was sampled following the established BATS methods manual (Knap et al., 1997); during BIOS-SCOPE process cruises, multiple depth profiles for the same parameters were sampled over 3–5 days.

**TABLE 1:** Spatial and temporal station information and sampling frequency for this study (x indicates sample collected and analyzed) for this study.

Time	Cruise	Station	DOC	TDAA	Metabolites	BA	BP	DNA
July 2016	BIOS-SCOPE AE1614	HS	X	x	x	x	x	x
July 2016	BATS 10326	BATS				x		x
August 2016	BATS 10327	BATS	X			x		x
September 2016	BIOS-SCOPE AE1620	HS	X	x	x	x	x	x
September 2016	BATS 10328	BATS	X			x		x
October 2016	BATS 10329	BATS	X	x		x		x
November 2016	BATS 10330	BATS	X		x	x		x
December 2016	BATS 10331	BATS	X	x		x		x
January 2017	BATS 10332	BATS	X		x	x		x
February 2017	BATS 10333	BATS	X	x		x		x
April 2017	BIOS-SCOPE AE1703	BATS	X	x	x	x	x	x
April 2017	BATS 10334	BATS	X			x		x
April 2017	BATS 20334	BATS	X			x		x
May 2017	BATS 10335	BATS	X	x	x	x		x
May 2017	BATS 20335	BATS				x		x
June 2017	BATS 10336	BATS		x		x		x
July 2017	BIOS-SCOPE AE1712	BATS	X	x	x	x	x	x
July 2017	BATS 10337	BATS	X			x		x
August 2017	BATS 10338	BATS	X	x		x		x
September 2017	BATS 10339	BATS	X		x	x		x

*Samples are from two stations: Hydrostation S (HS, 32°10′ N, 64°30′ W) and Bermuda Atlantic Time-series Study site (BATS, 31°40′ N, 64°10′ W). DOC: dissolved organic carbon, TDAA: total dissolved amino acid, BA: bacterioplankton abundance, BP: bacterial production.*

A Slocum glider was deployed in the vicinity (within a few kilometers) of the hydrostations from July 1st 2016 to August 4th 2016 and from January 6th 2017 to September 7th 2017. The glider was equipped with a Seabird pumped CTD, Aanderaa optode (model 4,831), and Wet Labs ECO chlorophyll fluorometer and backscatter optical sensors; and acquired measurements from the surface to either 500 or 900 m providing continuous profiles at intervals of 1–1.5 h. All sensors were annually serviced and calibrated by the manufacturer. Post-mission calibrations and corrections were applied to the sensors in accord with established practices and procedures (Allen et al., 2018; Woo and Gourcuff, 2021). CTD data were corrected for pressure bias, sensor lag, thermal mass inertia (Garau et al., 2011), spikes, and conductivity offset/drift (by comparison to collocated ship-based CTD casts). The oxygen data were corrected for sensor response time (Bittig et al., 2018), pressure, temperature, and salinity effects (Uchida et al., 2008), and offset (by comparison to ship-based CTD casts). Chlorophyll fluorescence was corrected for quenching by comparing daytime profiles to adjacent nighttime profiles to determine the depth of quenching and unbiased values near the surface. Factory-determined scaling factors were applied to both fluorescence and backscatter measurements to produce relative fluorescence units (RFU) and relative backscatter units (RBU). Within each glider mission, the profiles were evaluated and corrected for linear drift. The mission data were intercalibrated by aligning the profile minima onto common RFU and RBU values, respectively.

### Dissolved Organic Carbon

During BIOS-SCOPE cruises, seawater for DOC measurements was filtered inline through precombusted (450°C) 47 mm GF/F filters, packed in polycarbonate filter holders attached directly to Niskin-type sampling bottles, and collected into precombusted (450°C) 40 mL borosilicate glass vials with polytetrafluoroethylene (PTFE) coated silicone septa. All DOC samples were acidified with 4N HCl to pH ∼3. During monthly BATS cruises, total organic carbon (TOC) samples were collected with the same protocol as DOC but without filtration. Current analyses of bulk TOC and DOC in oligotrophic Sargasso Sea seawater are generally indistinguishable based on current instrument precision, especially in the subeuphotic realm (Supplementary Figure 1), thus TOC and DOC data in the Sargasso Sea were pooled together and presented as DOC hereafter. DOC concentrations were analyzed, referenced, and standardized using high-temperature combustion (HTC) on a modified TOC-V or TOC-L analyzer (Shimadzu) according to Carlson et al. (2010), with a precision of ∼1 μmol L^–1^ or CV of 2–3%. Daily reference water was calibrated with consensus reference material (CRM) from D. Hansell (University of Miami).

### Total Dissolved Amino Acids

Seawater for TDAA analysis was filtered through precombusted (450°C) 47 mm GF/F filters into either 40 mL EPA glass vials or 60 mL high-density polyethylene (HDPE) bottles and stored frozen at −20°C. Triplicate TDAA samples were hydrolyzed by 6N HCl (with 1% 12 mmol L^–1^ ascorbic acid to prevent oxidation of amino acids by nitrate) under nitrogen at 110°C for 20 h. Hydrolysate was then filtered through combusted quartz wool and neutralized under nitrogen via evaporation (Parsons et al., 1984). Amino acids were derivatized with o-phthaldialdehyde and analyzed by high-performance liquid chromatography (HPLC, Dionex ICS 5000+) equipped with a fluorescence detector (ThermoFisher RF2000, Ex = 330 nm, Em = 418 nm) following the established protocol with modifications (Lindroth and Mopper, 1979; Kaiser and Benner, 2009; Liu et al., 2013). In brief, the mobile phase consisted of solvent A of 50 mmol L^–1^ sodium acetate (adjusted to pH 5.7) and solvent B was 100% methanol (HPLC grade). Samples were pumped at a flow rate of 0.9 mL min^–1^ through a C18 column (Acclaim^TM^ 120, 5 μm, 120 Å, 4.6 × 250 mm) maintained at 17°C. Gradient program followed as: within 4 min, solvent B increased from 23 to 29%, and then increased to 44% solvent B at 20 min and 60% solvent B at 33 min; solvent B kept at 60% for 7 min, and increased to 77% in the next 8 min; solvent B further went up to 100% at 53 min, kept at 100% for 5 min, and then decreased to 23% at 60 min. The phenylalanine (Phe) peak occasionally co-eluted with a broad unidentified peak within our seawater samples. Due to its low relative abundance (<3% of TDAA) in oligotrophic Sargasso seawater (Keil and Kirchman, 1999; Kaiser and Benner, 2009), Phe was excluded from further analysis. The coefficient of variation (CV) was between 10 and 20% for replicate TDAA measurements. Outliers with > 20% variance among replicates were excluded (less than 10% in each batch were removed). TDAA-C was the sum of all amino acids in carbon units calculated based on the molecular formula and concentration of individual amino acids.

### Dissolved Metabolites

Seawater (4–10 L) was collected from Niskin-type sampling bottles, filtered through a 0.2 μm Omnipore filter (EMD Millipore), and acidified with concentrated hydrochloric acid. Organic metabolites were extracted from the acidified filtrate using solid phase extraction (SPE) with Agilent Bond Elut PPL cartridges as previously described (Dittmar et al., 2008; Longnecker, 2015). Methanol extracts were evaporated to near dryness using a Vacufuge (Eppendorf) and reconstituted in 250 μL of 95:5 (v/v) water/acetonitrile with isotopically labeled standards. Samples were analyzed using ultra high-performance liquid chromatography (Accela Open Autosampler and Accela 1250 Pump, Thermo Scientific) coupled to a heated electrospray ionization source (H-ESI) and a triple quadrupole mass spectrometer (TSQ Vantage, Thermo Scientific) operated under selected reaction monitoring (SRM) mode (Kido Soule et al., 2015). Separation was performed at 40°C on a reverse phase column (Waters Aquity HSS T3, 2.1 × 100 mm, 1.8 μm) equipped with a Vanguard pre-column. Mobile phase A was 0.1% formic acid in water and mobile phase B was 0.1% formic acid in acetonitrile. The flow rate was maintained at 0.5 mL min^–1^. The gradient began at 1% B for 1 min, increased to 15% B from 1 to 3 min, then increased to 50% B from 3 to 6 min, and then increased to 95% B from 6 to 9 min. The mobile phase was maintained at 95% B until 10 min and then decreased to 1% B from 10 to 10.2 min and held at 1% B for the remainder (12 min total run time). Samples were run in both positive and negative ion modes using a 5 μL injection for each mode. Raw data files were converted to mzML format prior to processing with MAVEN (Melamud et al., 2010; Clasquin et al., 2012) to identify and integrate peaks for all samples and standards. Peaks were identified by their unique retention time, precursor m/z, and product m/z. Two SRM transitions were monitored for quantification and confirmation. Metabolite concentrations were calculated using a standard curve of at least five points and were corrected for the efficiency of the solid phase extraction step using the data available from Johnson et al. (2017) and unpublished data for compounds not included in Johnson et al. (2017) (Supplementary Table 2).

### Bacterioplankton Abundance

Ten to forty mL of seawater was collected in sterile tubes (Falcon^TM^), fixed with 0.2 μm filtered formalin (1% final concentration), and stored at 4°C. Slides for microscopy were processed within 48 h of collection or stored at −80°C until processing in the shore-based laboratory. Seawater was filtered onto Irgalan Black stained 0.2 μm polycarbonate filters under gentle vacuum (∼100 mm Hg) and cells were stained with 5 μg mL^–1^ 4′,6′-diamidino-2-phenylindole dihydrochloride (DAPI, Sigma-Aldrich). Filters were mounted onto slides with Resolve immersion oil (high viscosity) and enumerated under ultraviolet excitation using an epifluorescence microscope (Olympus AX70 or Olympus BX51) at 1,000 × magnification (Porter and Feig, 1980). At least 10 fields or 500 cells per slide were counted. As this method cannot differentiate between bacteria and archaea, we refer to counted cells as bacterioplankton henceforth.

### ^3^H- Leucine Incorporation

^3^H-leucine incorporation rates are often used as a proxy for heterotrophic bacterioplankton production rates (Kirchman et al., 1985). Duplicate 1.6 mL seawater samples were amended with 20 nmol L^–1^ of ^3^H-leucine (Perkin Elmer, specific activity > 50 Ci mmol^–1^), and incubated at close to in situ temperatures for 3–4 h. Incubations were terminated by 100% trichloroacetic acid (TCA, 6% final concentration) and extracted with 5% TCA and 80% ethanol via the microcentrifuge method (Smith and Azam, 1992; Baetge et al., 2021). Seawater plus ^3^H-leucine but with 100% TCA (6% final concentration) added at the initiation of the incubation served as killed controls and followed the same incubation and extraction steps. Samples were mixed with 1.5 mL Ultima Gold (Perkin Elmer LLC), left for at least 2 h, and measured in a liquid scintillation counter.

### 16S rRNA Amplicon Sequencing

Four liters of seawater were filtered onto 0.2 μm Sterivex filters (polyethersulfone membrane, Millipore, Burlington, MA, United States) and stored in sucrose lysis buffer at −80°C. DNA was extracted using a phenol-chloroform protocol (Giovannoni et al., 1996) and amplified with V1-V2 primer 27F (5′-AGAGTTTGATCNTGGCTCAG-3′) and 338RPL (5′-GCWGCCWCCCGTAGGWGT-3′) linked to “general” Illumina overhang adapters (Bolaños et al., 2020). Libraries were pooled in equimolar concentrations and sequenced using 2 × 250 Pair-End lanes with a MiSeq Reagent Kit v2 at the Center for Genome Research and Biocomputing [CGRB, now known as Center for Quantitative Life Sciences (CQLS)] at Oregon State University. The 16S rRNA sequences were trimmed and dereplicated, chimeras were removed and the resulting sequences were assigned to amplicon sequence variant (ASV) taxonomy using the DADA2 R package, version 1.2 (Callahan et al., 2016) and Silva database (version 123). For the unassigned ASV sequences, they were first BLAST searched against a non-redundant 16S rRNA sequence database and second against the full non-redundant database, and then assigned to the top BLAST hit (Altschul et al., 1990, 1997; Camacho et al., 2009). SAR11 and SAR202 ASVs were further run through the PhyloAssigner program (Vergin et al., 2013a) using customized phylogenetic trees for fine-scale phylogenetic assignment within these clades (Bolaños et al., 2021).

### Data and Statistical Analysis

#### Mixed Layer Depth

Mixed layer depth (MLD) was defined as the depth where density (sigma-theta) decreased by an increment determined by the thermal expansion coefficient (αΔT) using a ΔT of 0.2°C (Sprintall and Tomczak, 1992). The depth of the chlorophyll maximum (DCM) was determined from fluorometer profiles. The upper and lower boundaries of the DCM layer (DCML) are defined as the depths where chlorophyll fluorescence is 35% of the maximum value. Four seasons were defined by the relative positions of the MLD and DCM or DCML determined from CTD and daily-averaged glider profiles (when the glider was not present at the station, CTD data were used): (1) The Mixed season began when the MLD first reached below the top of the DCML and ended when the MLD rapidly shoaled above the DCM; (2) Spring transition began at this point and ended when stratified conditions were established and the MLD remained above the top of the DCML; (3) the Stratified season spanned the period during which the MLD was consistently shallower than the top of the DCML; (4) Fall transition began when the MLD first reached below the top of the DCML. Apparent oxygen utilization (AOU), defined as the difference between the saturated oxygen concentration and the observed oxygen concentrations, was calculated based on CTD depth, temperature, salinity, and dissolved oxygen concentrations using Ocean Data View (ODV, version 5.4.0) (Schlitzer, 2021).

#### Dissolved Organic Matter Degradation Index

The DOM degradation index (DI) was calculated from the TDAA data following Dauwe et al. (1999) as:


$$\text{DI}=\sum_{\text{i}}\left[\frac{{\text{var}}_{\text{i}}-\text{AVG}\,{\text{var}}_{\text{i}}}{\text{STD}\,{\text{var}}_{\text{i}}}\right]\times \text{fac}.{\text{coef}}_{\text{i}}$$

where var_*i*_ is the mole percentage of amino acid i, AVG var_*i*_ and STD var_*i*_ are its mean and standard deviation, and fac.coef_*i*_ is the factor coefficient for amino acid i (Kaiser and Benner, 2009); a lower DI value indicates more diagenetically altered DOM. Significant differences in biogeochemical variables between periods of deep mixing and other times (including spring transition, stratified, and fall transition) were evaluated with unpaired *t*-tests (*p* < 0.05). Spearman correlation analysis with biogeochemical data was conducted for the respective mixing and non-mixing time periods. Mean values over water depths for concentration-type variables, including DOC, TDAA, and BA, were integrated over target depths (i.e., euphotic zone, 0–120 m; or upper mesopelagic zone, 120–300 m) and then depth normalized for each depth zone; while mean values for other variables, such as temperature, DI, AOU, and 16S amplicon relative abundance (see below), were calculated as average of values at discrete depths.

#### Controls on the Concentrations of Metabolites, Dissolved Organic Carbon, Total Dissolved Amino Acids, and Bacterioplankton Abundance in the Mixed Layer

Changes in the concentrations of targeted dissolved metabolites, DOC, TDAA-C, and BA can result from conservative dilution due to mixing, or production or loss due to other biotic and abiotic processes. Below is an example of how we assessed whether changes were attributed to mixing or other processes for metabolites, but the same calculations were performed for DOC, TDAA-C, and BA. The metabolite concentrations at a given time were first integrated to the mixed layer depth at the next time point. This integrated value was then compared to the metabolite at the next time point, which had been integrated to the mixed layer depth of this subsequent time point (Supplementary Figure 2A). If the integrated value of the metabolite were equal between two consecutive time points, then the variability in the metabolite concentrations at any given depth in the mixed layer between time points was deemed a function of mixing. When the integrated values to the MLD differed between time points, the changes were due to production or consumption of the metabolite. To consider if this production or loss was significant, the distance of each point representing the targeted metabolite to the 1:1 line that corresponds to mixing was calculated (Supplementary Figure 2B). This distance was then converted to a standardized z-score (distance of one metabolite at one time point minus the mean of that metabolite at all-time points and then divided by the standard deviation of distance of that metabolite at all-time points) so that the mean for each metabolite was 0 and the standard deviation was 1. Calculated z-scores were fit to a normal distribution using pd = fitdist(Z(:), “normal”) in MATLAB (version 2019b) to compare with the inverse cumulative distribution function of those z-scores fitted to a normal distribution using icdf (pd, 0.95). Any metabolite at a time point that was greater than the value calculated in the inverse cumulative distribution function was treated as a significant production or loss. The multivariate homogeneity of groups dispersion (Anderson, 2006) was calculated using “betadisper” in R and showed that the April 2017 samples were different from all other time points.

#### 16S rRNA Amplicon Data Analysis

Non-metric multidimensional scaling (NMDS) of the bacterial community structure for the entire record between 2016 and 2017 was based on Bray-Curtis dissimilarities estimated using phyloseq (McMurdie and Holmes, 2013) and vegan (Oksanen et al., 2007) and plotted with ggplot2 package in R (Wickham, 2009) (R version 3.3.2). Hierarchical clustering analysis was conducted using Simprof analysis (α = 0.05) with clustsig package in R (Whitaker and Christman, 2015). ASVs that are not classified at the class level or assigned as chloroplasts were excluded for the following analysis. We used two criteria to define ASVs that were enriched in the upper mesopelagic (120–300 m) during or shortly following convective mixing: (1) the maximal mean relative abundance of the ASV between 120 and 300 m occurred during the April 2017–July 2017 period; (2) the mean relative abundance of the ASV within the mixed layer in April 2017 (maximal mixing) was greater than that in November 2016 (before mixing), thus excluding ASVs with changes of mean relative abundance solely due to conservative mixing. Among mesopelagic ASVs discussed above, to further identify heterotrophic ASVs in response to DOM export, we excluded autotrophic cyanobacteria and then did a cross-correlation functions (CCF) analysis between the mean relative abundance of an ASV and the monthly averaged mean 120–300 m DOC or TDAA C. CCF analysis is a correlation analysis between two time-series datasets. It provides not only the correlation coefficient between two variables, but also lags of one variable that may be predictors of the other. For instance, negative lags of ASVs relative to DOC indicate DOC is the predictor of ASV, whereas positive lags of ASVs relative to DOC indicate vice versa. ASVs with significant positive correlation and negative lagged months relative to DOC and/or TDAA C (indicating changes of DOC and/or TDAA lead to changes in the ASV) were the focus of this study.

### Data Deposition

BIOS-SCOPE cruise data are deposited in the Biological and Chemical Oceanography Data Management Office (BCO-DMO) at http://lod.bco-dmo.org/id/dataset/861266, and BATS cruise data are deposited at http://lod.bco-dmo.org/id/dataset/3782 and http://bats.bios.edu/bats-data/. 16S amplicon sequences are deposited in the National Center for Biotechnology Information (NCBI) Sequence Read Archive (SRA) under project number PRJNA769790. Targeted metabolomics data for this project are deposited at MetaboLights^1^ as study accession number MTBLS2356.

## Results and Discussion

### Seasonal Change of Chemical Parameters in the Sargasso Sea

#### Dynamics of Dissolved Organic Carbon

**FIGURE 1:**
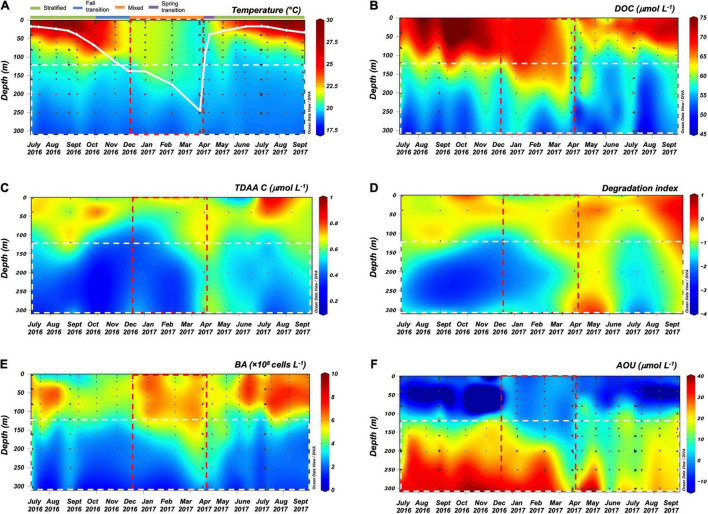
ODV contour plots of **(A)** temperature (solid white line represents monthly maximum mixed layer depth (MLD) and the colored bar on top represents seasons), **(B)** dissolved organic carbon (DOC) concentration, **(C)** total dissolved amino acid carbon (TDAA C) concentration, **(D)** degradation index (DI) from TDAA, **(E)** bacterioplankton abundance (BA), and **(F)** apparent oxygen utilization (AOU) from July 2016 to September 2017 over the surface 300 m of northwestern Sargasso Sea. Red dashed rectangle indicates the general timing of deep convective mixing, and white dashed rectangle at 120–300 m defines the upper mesopelagic zone.

The seasonal variability of dissolved inorganic nutrients, chlorophyll fluorescence, particulate organic matter (data not shown, BATS core data)^2^,^3^ and dissolved organic matter observed during this study are consistent with seasonal cycles of hydrography and organic carbon dynamics previously observed in this region (Hansell and Carlson, 2001; Steinberg et al., 2001; Lomas et al., 2013). During winter convective mixing from December 2016 to April 2017, the MLD extended beyond the euphotic zone (0–120 m) into the upper mesopelagic zone (120–300 m) to depths as great as 242 m (April 2017) (Figure 1A, euphotic zone and upper mesopelagic zone separated by white dashed line). Winter convective mixing entrains inorganic macronutrients from depth into the euphotic zone, supporting the annual phytoplankton bloom and buildup of chlorophyll *a* and particulate organic matter in the surface following mixing (Lomas et al., 2013). DOC in the surface 0–120 m accumulated at the rate of 24 nmol L^–1^ d^–1^ as the water column began to stratify in April, and this DOC production continued until August (Table 2), then it persisted at relatively constant concentrations from summer into late fall (Figure 1B; Carlson et al., 1994; Hansell and Carlson, 2001; Goldberg et al., 2009).

**TABLE 2:** Mean change in DOC and TDAA C concentrations and their net production rates in the euphotic zone (0–120 m) and net removal rates in the upper mesopelagic zone (120–300 m) from April 2017 (time of max MLD) to August 2017.

Depth	Month	Duration (days)	ΔDOC (μmol L^–1^)	DOC rate (nmol L^–1^ d^–1^)	*R* ^2^	*n*	Duration (days)	ΔTDAA C (μmol L^–1^)	TDAA C rate (nmol L^–1^ d^–1^)	*R* ^2^	*n*
0–120 m production	April–August	136	3.2	24	0.43	9	136	0.03	0.2	0.1	6
120–300 m consumption	April–August	136	4.0	30	0.49	9	136	0.10	0.7	0.6	6

*Rates were derived from linear regression with R^2^ and number (n) of data points shown.*

**FIGURE 2:**
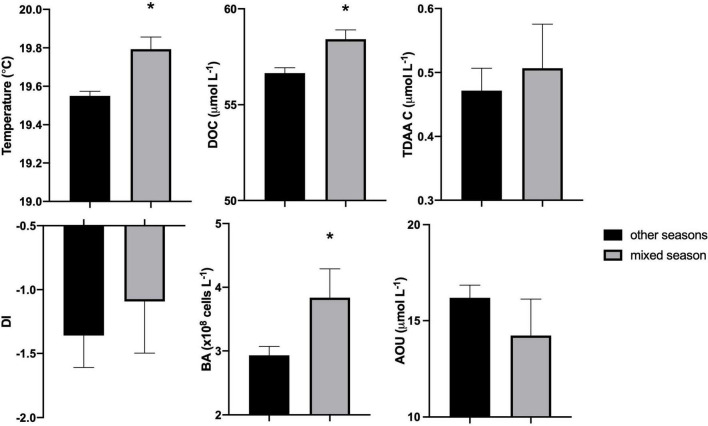
Comparison of upper mesopelagic (120–300 m) mean temperature, dissolved organic carbon (DOC), total dissolved amino acid carbon (TDAA C), degradation index (DI), bacterioplankton abundance (BA), and apparent oxygen utilization (AOU) between mixed season and other seasons (including spring transition, stratified, and fall transition) from July 2016 to September 2017. Bars represent mean with standard error and asterisk (*) designates significant differences between the periods (unpaired *t*-test, *p* < 0.05).

Despite being recently produced, much of the surface accumulated DOC resists or escapes rapid microbial degradation by the euphotic zone heterotrophic microbial community on time scales of days to weeks (Carlson et al., 2002, 2004; Liu et al., 2020b). This DOC is redistributed during the period of winter deep convective mixing, thereby reducing surface DOC concentrations and enriching mesopelagic concentrations (Figures 1A,B, Supplementary Figure 3A). Mean DOC concentrations in the upper mesopelagic (120–300 m) were significantly greater during the mixing period compared to those in the non-mixing period (unpaired *t*-test, *p* < 0.05) (Figure 2). Based on these changes in the mesopelagic zone, it is estimated that 0.60 mol m^–2^ yr^–1^ DOC was exported from the surface to the 120–300 m zone due to winter convective mixing. This estimate is comparable to previously reported values of DOC export suggesting that physical mixing of dissolved organic matter is an important export pathway in this region, which can be greater than measured particle export (Carlson et al., 1994; Michaels et al., 1994; Hansell and Carlson, 2001). Other processes such as solubilization of sinking POC flux (Cho and Azam, 1988) and DOC production *via* vertical migrators (Steinberg et al., 2002) are potential sources of DOC in the mesopelagic, however, redistribution of surface accumulated DOC *via* convection mixing is considered the dominant source of DOC to the mesopelagic during periods of deep mixing at this site (Carlson et al., 1994; Hansell and Carlson, 2001).

Upon restratification of the upper water column, a portion of the DOC that was exported to the mesopelagic was trapped and removed at a rate of 30 nmol L^–1^ d^–1^ (April to August) (Figure 1B and Table 2). We attribute the mesopelagic DOC removal to microbial remineralization. Why DOC accumulates and resists microbial degradation in the surface but becomes bioavailable in the mesopelagic remains unresolved. However, previous work has suggested that macronutrient availability (Cotner et al., 1997; Obernosterer et al., 2003), quality of the recently exported DOC (Goldberg et al., 2009), and the heterotrophic microbial community capable of responding to semi-labile/recalcitrant compounds at depth (Carlson et al., 2004; Letscher et al., 2015; Liu et al., 2020a; Saw et al., 2020) all interact to control mesopelagic DOC removal rates.

#### Dynamics of Total Dissolved Amino Acid

Our study provides the first seasonal dataset of TDAA in the Sargasso Sea revealing how DOM quality changes over depth and time. The TDAA-C represents < 1.6% of DOC in the Sargasso Sea (Figure 1C) but remains a useful index of DOM quality because its variability is influenced by both biological production and degradation processes. As such, TDAA can be used to trace potential DOM origins and degradation state (Cowie and Hedges, 1994). TDAA composition in the Sargasso Sea was dominated by glycine, alanine, aspartic acid, glutamic acid, and serine (Supplementary Table 1), consistent with previous studies of amino acid data in this region (Keil and Kirchman, 1999; Kaiser and Benner, 2009). The enriched TDAA-C and higher DI in the upper mesopelagic zone (120–300 m) following mixing (Figures 1C,D, 2), relative to the fully stratified periods, indicate that relatively less diagenetically altered DOM was exported from surface to depth within the mixing time frame (Figure 1D). Coincident with the deepest MLD in April, TDAA-C concentrations were enriched throughout the upper 300 m by 41% compared to the earlier stratified period (e.g., October 2016) and its production was significant in the mixed layer (Supplementary Figure 3B), suggesting net production of TDAA-C during the mixing period; a period that coincides with enhanced biological production for this site (Steinberg et al., 2001; Lomas et al., 2013). The elevated TDAA-C concentrations observed in the upper mesopelagic zone (120–300 m) are interpreted as a combination of both recently produced TDAA and export of previously accumulated surface TDAA to depth during convective mixing.

In the present study, TDAA-C accumulated in the upper 120 m at a rate of 0.2 nmol L^–1^ d^–1^ shortly following maximal deep mixing and when the MLD shoaled to above 120 m, reaching a maximum of 1.04 μmol L^–1^ in the euphotic zone by July (Table 2). During the stratified periods, the TDAA concentrations increased in the upper 120 m where photoautotrophy and subsequent food web processing (i.e., grazing, viral infection, or POM solubilization) are potential sources of new TDAA production. DOM from phytoplankton is largely composed of labile and semi-labile compounds such as carbohydrates and TDAA, which can account for 30–50 and 10–30% of exudate DOC, respectively (Sieburth et al., 1977; Burney et al., 1982; Tada et al., 2017). Zooplankton grazing and egestion is a potential source of TDAA release with previous studies demonstrating that 4–48% of total nitrogen excreted by zooplankton is in the form of TDAA (Nagata and Kirchman, 1991; Steinberg and Saba, 2008). In addition, Smith et al. (1992) found that 0.5–24 mg m^–2^ d^–1^ nitrogen can be released as DCAA from marine aggregates in the mixed layer of the Southern California Bight.

In contrast to the accumulation in the euphotic zone, TDAA was removed at a rate of 0.7 nmol L^–1^ d^–1^ in the upper mesopelagic following deep mixing (Table 2). Uptake of amino acids can account for 10–20% of bacterial carbon demand and 5–50% of bacterial nitrogen demand (Suttle et al., 1991; Keil and Kirchman, 1999; Kirchman, 2000), indicative of their active role in biological metabolism. Bacteria consume amino acids as nutrients and use them for anabolism of macromolecules including proteins and fatty acids (Cottrell and Kirchman, 2000; Aepfler et al., 2019). While apparently resistant to rapid microbial degradation, the DOM exported from euphotic to mesopelagic layers in the Sargasso Sea during winter mixing was less diagenetically altered compared to mesopelagic DOM under stratified conditions, as indicated by TDAA degradation index. Thus, exported DOM was more likely to be predominantly semi-labile and/or semi-refractory DOM (Carlson, 2002; Carlson et al., 2011; Hansell, 2013). This temporal and vertical pattern in the TDAA is consistent with the seasonality of DOM quality revealed from DCNS data from this region (Goldberg et al., 2009). The enhanced DCNS concentrations and relative change in DCNS composition within the mesopelagic followed the export of surface-accumulated DCNS to depth during winter convective mixing, and DCNS concentration, % DCNS yield, and neutral sugar composition changed over time once isolated in the mesopelagic zone.

#### Temporal and Spatial Variability of Dissolved Metabolites

**FIGURE 3:**
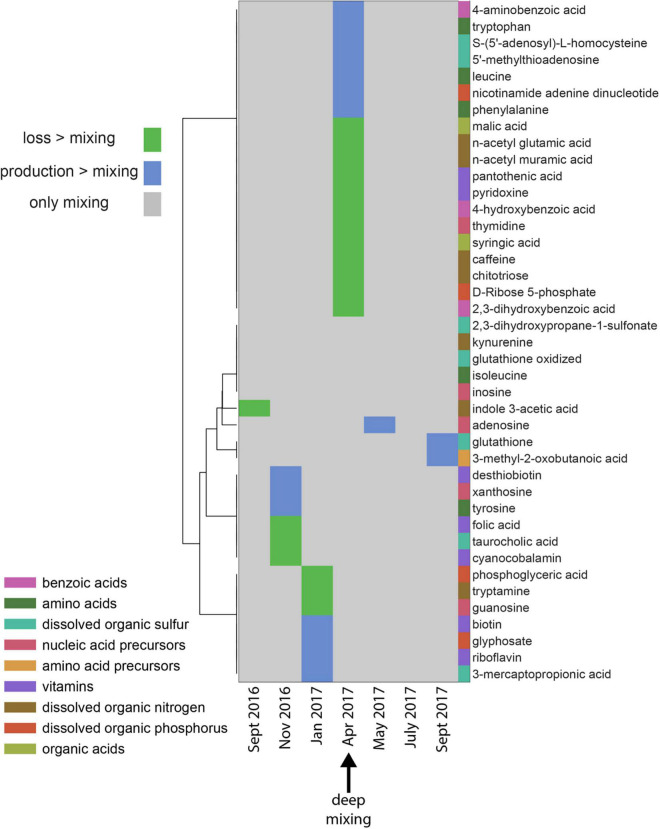
Patterns of production or loss of metabolites in the mixed layer over the seasonal cycle. Green areas indicate the net loss of a metabolite relative to conservative mixing in that time-period compared to the previous month, blue areas indicate net production of a metabolite relative to conservative mixing, while gray areas indicate metabolite concentration changes were solely due to mixing. Metabolite categories are shown as different colors besides the metabolite names. These categories are not exclusive; Supplementary Table 2 lists the categories shown here as well as additional categories. The statistical methods (described in methods) determined that the metabolite pattern in April (max mixing) was different from other time periods.

In addition to bulk DOM and TDAA, a variety of targeted dissolved metabolites revealed unique seasonal patterns (Figure 3, see Supplementary Figure 4 for depth profiles of all targeted metabolites over the season). In April 2017 when MLD reached the annual maximum, a group of diverse metabolites demonstrated positive or negative changes in the mixed layer from the previous sampling month that exceeded changes attributed solely to abiotic mixing processes. Free amino acids, including leucine, phenylalanine, and tryptophan, were produced and accumulated throughout the mixed layer during convective mixing. A possible source of these metabolites is the *de novo* synthesis of amino acids by bacteria from inorganic nitrogen entrained to the surface waters during deep mixing and subsequent release of those amino acids into the dissolved pool (Maki et al., 2014; Yamaguchi et al., 2017).

The vitamins pyridoxine (vitamin B_6_) and pantothenic acid (vitamin B_5_) were depleted in April relative to January (Figure 3). Vitamin B complexes, present at undetectable to picomolar concentrations in seawater (Sañudo-Wilhelmy et al., 2014; Suffridge et al., 2017; Joglar et al., 2021), are essential coenzymes for eukaryotic phytoplankton such as species in Chlorophyta, Heterokontophyta, and Dianophyta (Croft et al., 2005; Sañudo-Wilhelmy et al., 2014). It has also been shown that auxotrophic phytoplankton such as the diatom *Thalassiosira pseudonana* and the coccolithophore *Emiliania huxleyi* are often vitamin limited (Bertrand and Allen, 2012). We hypothesize that microbes scavenge pyridoxine and pantothenic acid to meet their metabolic demands during periods of deep mixing.

Benzoic acids and nucleic acid precursors, including intermediate products (i.e., 4-aminobenzoic acid, tryptophan, phenylalanine, and 4-hydroxybenzoic acid) formed in the shikimate pathway (Averesch and Krömer, 2018), revealed a disparate response with some intermediates being produced and others being lost during mixing. The shikimate pathway is the central metabolic pathway in plants and microorganisms for the synthesis of aromatic amino acids and folates (Wilson et al., 1998; Herrmann and Weaver, 1999). Metabolite variability such as the production of 4-aminobenzoic acid, tryptophan, phenylalanine, and the loss of 4-hydroxybenzoic acid observed during mixing in the Sargasso Sea indicates a nuanced microbial response to the mixing event. Various DON compounds, such as n-acetyl glutamic acid, n-acetyl muramic acid, chitotriose, and caffeine, were depleted during deep convective mixing in April, indicating these N-enriched labile compounds are preferentially removed.

The dissolved organic sulfur compound, 5-methylthioadenosine (MTA), was also elevated during the period of maximal mixing. MTA plays an important role in bacterial metabolic processes such as methylation, polyamine biosynthesis, regulation of gene expression, and cell proliferation (Avila et al., 2004). Furthermore, a meridional study of metabolites revealed higher MTA concentrations in the surface ocean of the North Atlantic compared to the south Atlantic Ocean. This observation is hypothesized to be a reflection of differences in the metabolic pathways of phytoplankton known to produce MTA (Johnson et al., 2021, in revision). While extracellular release of MTA is limited in the cyanobacterium *Synechococcus* (Fiore et al., 2015), it was identified as an extracellular metabolite in cultures of the heterotrophic bacteria *Ruegeria pomeroyi* (Johnson et al., 2016). While the exact mechanisms controlling MTA accumulation are unclear, the mere presence of MTA in the water column at BATS indicates that there is an imbalance between consumption of MTA and enhanced production by microbial activity during the mixing period.

### Seasonality of Mesopelagic Bacterioplankton Dynamics and Community Structure in Response to Deep Convective Mixing and Dissolved Organic Matter Export

**FIGURE 4:**
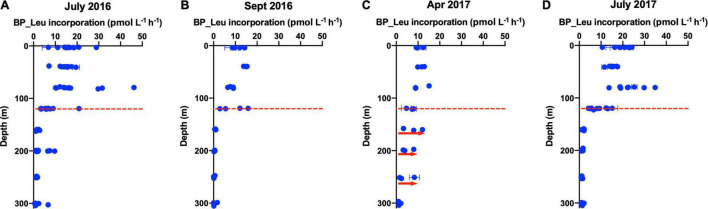
Vertical profile of ^3^H-Leucine incorporation rates, a proxy for bacterial production (BP), over the surface 300 m during cruises in **(A)** July 2016, **(B)** September 2016, **(C)** April 2017, and **(D)** July 2017. Data points represent the average of replicates and error bars are standard error. The red dashed line (120 m) separates the euphotic zone from the upper mesopelagic zone. Red arrows indicate enhancement of BP in the mesopelagic zone during mixing in April 2017 compared to other stratified periods.

During the period of deep convective mixing when MLD extended beyond the euphotic zone (i.e., below 120 m), bacterioplankton cell density increased within the upper mesopelagic zone (120–300 m) and persisted at elevated levels for weeks (Figure 1E). The mean BA in the mesopelagic zone (120–300 m) during the mixing period was significantly greater than during the stratified period (*t*-test, *p* = 0.0174) (Figure 2). Significant increase of BA in the mixed layer was observed in January relative to December, indicating this increase was not only due to conservative mixing (Supplementary Figure 3C). This seasonal pattern is consistent with historical data of bacterioplankton biomass in this region (Carlson et al., 1996; Steinberg et al., 2001). AOU in the upper mesopelagic zone decreased during mixing and increased afterward (Figures 1, 2). During the period when the MLD was deepest, the ^3^H-leucine incorporation rates, a proxy of BP, were three to five times greater in the mesopelagic compared to rates at the same depth during stratified periods (Figure 4). The enhanced ^3^H-leucine incorporation rate at depth during periods of deep mixing indicates that heterotrophic bacterioplankton were responding to the production or delivery of relatively less diagenetically altered organic matter to the mesopelagic zone. Significant correlations between upper mesopelagic mean temperature and DOC, temperature and BA, temperature and AOU, DOC and BA, DOC and AOU, and TDAA-C and DI were observed over this seasonal time-series (Table 3). Together, the data suggest that DOC export during convective mixing triggered the growth of heterotrophic bacterioplankton that were involved in the degradation of exported DOM (Figure 1). The exported DOM includes fresh and recently produced compounds as well as those that were resistant or escaped rapid remineralization by upper euphotic zone heterotrophic bacterioplankton populations.

**TABLE 3:** Correlation matrix (Spearman *r*-value) between 120 and 300 m mean temperature, DOC, TDAA C, DI, BA, and AOU over the seasonal cycle (July 2016 to September 2017) in the Sargasso Sea.

	Temperature	DOC	TDAA C	DI	BA	AOU
Temperature	1.00	**0.43**	0.07	0.21	**0.50**	**0.32**
DOC	**0**.**43**	1.00	–0.13	–0.11	**0.35**	**−0.34**
TDAA C	0.07	–0.13	1.00	**0.86**	0.22	–0.13
DI	0.21	–0.11	**0.86**	1.00	0.32	0.03
BA	**0**.**50**	**0.35**	0.22	0.32	1.00	−0.06
AOU	**0.32**	**−0.34**	–0.13	0.03	–0.06	1.00

*Significant correlations (p < 0.05) are indicated as bold values.*

**FIGURE 5:**
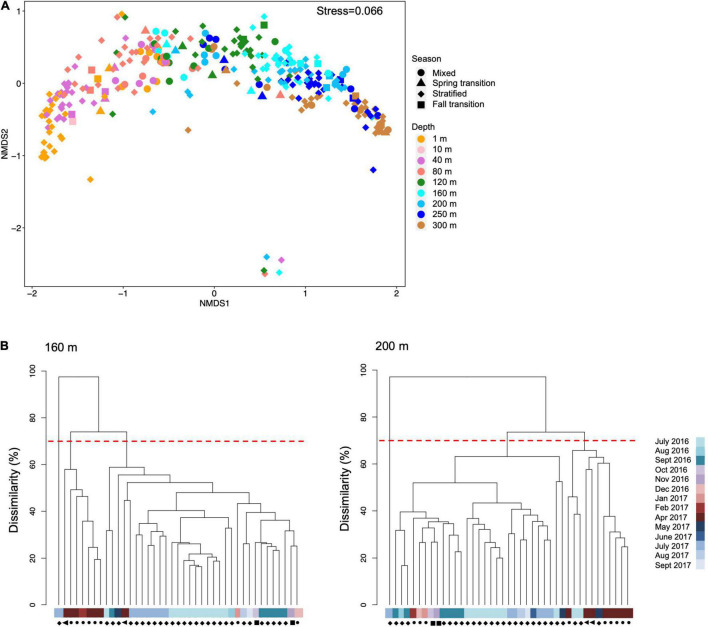
**(A)** Non-metric multi-dimensional scaling (NMDS) plot showing first two axes of all 16S amplicon sequence variants (ASVs) color coded by depths and shaped by seasons over July 2016 to Sept 2017. The five points in the bottom part of the NMDS plot came from the same cast in July 2017. We cannot explain the driver of these five outliers, nor could we identify a reason to remove them; thus, they are included in the plot. **(B)** Simprof cluster analysis of bacterial community structures at 160 and 200 m. Different clusters are shown at 70% dissimilarity threshold. Samples names are in color bar and symbols next to each sample name indicate season with symbol shapes as defined in **(A)**.

Bacterioplankton community structure based on 16S rRNA amplicon sequencing was clearly separated along NMDS axis 1 according to depth (Figure 5A). Vertical stratification of microbial taxa has been reported for numerous studies in the northwestern Sargasso Sea and elsewhere (Giovannoni et al., 1996; DeLong et al., 2006; Treusch et al., 2009; Vergin et al., 2013a). Bacterial community structure in the upper mesopelagic zone was more variable than in the surface ocean. During the highly mixed and spring transition periods at 160 m and 200 m, bacterial communities clustered as a distinct group from stratified and fall transition clusters (Figure 5B), suggesting mesopelagic bacterial communities were significantly altered by the mixing event.

**FIGURE 6:**
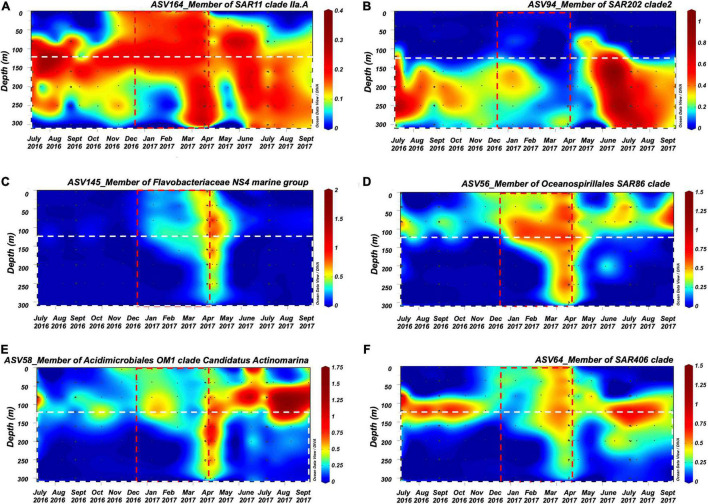
ODV contour plots of the ASV relative abundance (%) in the surface 300 m for bacterioplankton lineages in the **(A)** SAR11 clade, **(B)** SAR202 clade, **(C)** Flavobacteriaceae NS4 marine group, **(D)** SAR86 clade, **(E)** Acidimicrobiales OM1 clade, and **(F)** SAR406 clade that showed enrichment in the mesopelagic zone during or shortly after mixing events and that were significantly positively cross-correlated with DOC and/or TDAA C with negative lag within July 2016 to September 2017 time frame. Red dashed rectangle indicates convective mixing time frame during this time period and white dashed rectangle indicates 120–300 m of the upper mesopelagic zone.

**FIGURE 7:**
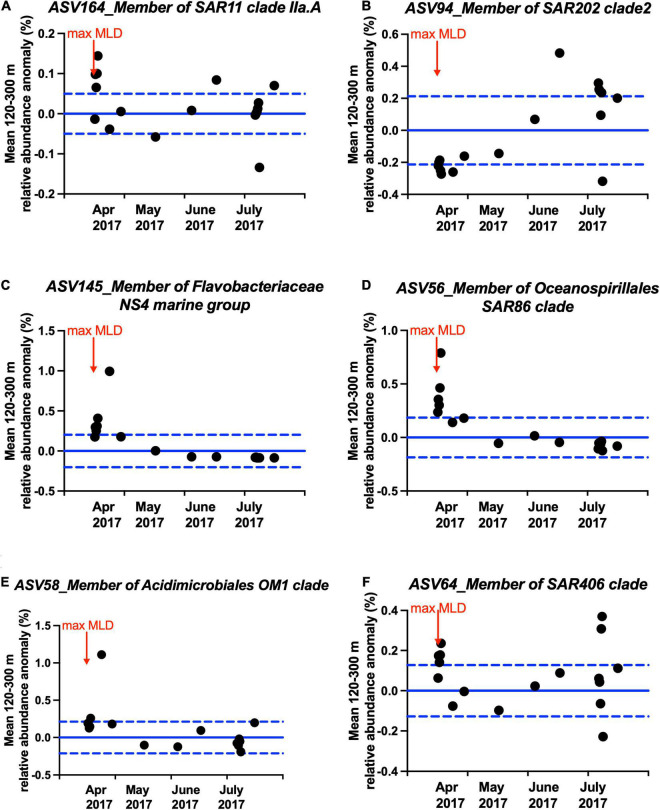
Anomaly between observed mean relative abundance between mesopelagic 120–300 m during and shortly following maximal mixing (April 2017 to July 2017) and mean mesopelagic 120–300 m relative abundance during the entire study period for representative ASVs in the **(A)** SAR11 clade, **(B)** SAR202 clade, **(C)** Flavobacteriaceae NS4 marine group, **(D)** SAR86 clade, **(E)** Acidimicrobiales OM1 clade, and **(F)** SAR406 clade as shown in Figure 6. Blue solid lines and dashed lines indicate zero and ± one standard deviation of all-time data, respectively. Data points above zero suggest enrichment of that ASV during or shortly after mixing. Arrow indicates max MLD time.

Multiple members of the bacterioplankton SAR11 clade, OCS116 clade, *Rhodospirillales*, *Rhodobacterales*, SAR116 clade, SAR202 clade, *Flavobacterales*, *Oceanospirillales*, *Salinisphaerales*, *Alteromonadales*, *Verrumicrobiae*, *Acidimicrobiales*, SAR406 clade, and SAR324 clade became enriched during or shortly following deep convective mixing and were significantly cross-correlated to mean DOC and TDAA-C concentrations in the upper mesopelagic zone with maximum correlation coefficient being reached with a 0–4 months lag depending on the bacterioplankton lineage (Figure 6, see Supplementary Table 3 and Supplementary Figure 5 for all enriched ASVs). The mean relative abundance of each of these lineages within the upper mesopelagic (120–300 m) was greatest during or following the maximal mixing period (April 2017–July 2017) compared to the average over all months between July 2016 and September 2017 (Figure 7). The temporal increase in the relative abundance of these specific lineages (Figure 6 and Supplementary Figure 5) together with enhanced BA and ^3^H-leucine incorporation rate in the mesopelagic zone during or following deep mixing suggest that the increased bacterial biomass and activity in the mesopelagic during mixing was not solely due to mixing of the surface population into the mesopelagic depths, but rather due to responses of specific bacteria at depth to the exported organic matter.

DOM exported to the mesopelagic *via* physical mixing contains a continuous spectrum of compounds with varying lability and corresponding myriad responses from bacterial taxa with different nutrition-acquisition strategies and effective niches (Fuhrman and Hagström, 2008; LaBrie et al., 2020; Liu et al., 2020a). Bacteria characterized as copiotrophs such as members of the *Rhodobacterales* and *Rhodospirillales* clades are often associated with elevated primary production (Gilbert et al., 2012; Buchan et al., 2014; Wilson et al., 2017). These data suggest that some ASVs in these groups respond to the vertical export flux of labile metabolites, such as carbohydrates, amino acids, and nucleotides released from primary producers (Figure 3). For example, members of the *Flavobacteriales* clade are specialists that can assimilate high molecular weight (HMW) DOM and are capable of assimilating large polysaccharides into their periplasmic space *via* “selfish” uptake pathways (Cottrell and Kirchman, 2000; Reintjes et al., 2017). The enhanced relative contribution of *Flavobacteriales* in the upper mesopelagic might indicate the export of HMW DOM during the deep mixing period. Similarly, growth of *Verrucomicrobiae*, which are often enriched in particle-associated communities and possess genes or enzymes for polysaccharide degradation (Vorobev et al., 2018; Duret et al., 2019; Sichert et al., 2020), is consistent with the export of carbohydrates from surface to mesopelagic zone by convective mixing as revealed in previous research in the Sargasso Sea (Goldberg et al., 2009).

It is noteworthy that a number of slow-growing organisms characterized as oligotrophs such as members of the SAR11 clade, the SAR202 clade, the OCS116 clade, the SAR86 clade, *Acidimicrobiales*, *Salinisphaerales*, the SAR406 clade, and the SAR324 clade became enriched over timescales of weeks to months. The various lineages reach ASV maxima at different times during or following convective mixing (Figure 6, Supplementary Figure 5, and Supplementary Table 3). The SAR11 and SAR86 clades diversify into multiple ecotypes that partition niche space over depth and season (Carlson et al., 2009; Dupont et al., 2012; Vergin et al., 2013a; Giovannoni, 2017). The variability in lagged response between SAR11 ASVs and DOC and/or TDAA-C indicates that there can be differences in SAR11 ecotypes’ response to alteration of DOM concentration and quality in the mesopelagic water. Members of the SAR202 clade, the OCS116 clade, *Acidimicrobiales*, and *Salinisphaerales* are involved in, or possess genes encoding enzymes for, degradation of more recalcitrant DOM (Goldman and Green, 2009; Mizuno et al., 2015; Landry et al., 2017; Rodríguez et al., 2018; Liu et al., 2020a,b). The increasing relative abundance of these lineages in the mesopelagic during and following convective mixing is consistent with the argument that they respond to the exported DOM of semi-labile and semi-refractory quality. Alternatively, the introduction of relatively less diagenetically altered DOM to the mesopelagic water during mixing (Figure 1D) might promote microbial utilization of resident recalcitrant DOM, through the priming effect (Carlson et al., 2002; Steen et al., 2016; Shen and Benner, 2018). Enrichment of members of the SAR406 clade during the period of deep mixing was consistent with its potential metabolic role in degrading complex carbohydrates and transporting amino acids across cell membranes as revealed from genomic analysis (Thrash et al., 2017). Our data also suggest that the members of the SAR324 clade, often characterized as chemoautotrophic bacteria (Swan et al., 2011), might be involved in seasonal DOM cycling either directly through heterotrophy behavior or indirectly through cross-feeding on byproduct during DOM cycling. This hypothesis is consistent with previous work showing that SAR324 possesses flexible mechanisms for DOC or DON utilization, including aromatic compound degradation and C1 metabolism (Swan et al., 2011; Sheik et al., 2014; Cao et al., 2016; Kitzinger et al., 2020).

Seasonal dynamics of microbial communities have been reported for multiple marine environments, and are often correlated to repeatable patterns of both abiotic and biotic environmental variables such as temperature, nutrient concentrations, day length, and primary production (Fuhrman et al., 2006; Caporaso et al., 2012; Gilbert et al., 2012; Bunse and Pinhassi, 2017; Mena et al., 2020). Terminal restriction fragment length polymorphism (T-RFLP) and 454 pyrosequencing data from previous studies at BATS demonstrated that the seasonality of water column mixing and stratification is an important factor controlling bacterial community composition. For example, specific lineages within the OCS116, SAR11, and *Actinobacteria* (in which *Acidimicrobiales* belong) clades exhibit the strongest increases in relative abundance in the upper mesopelagic zone from the stratified period to the period following convective mixing (Morris et al., 2005; Carlson et al., 2009; Treusch et al., 2009; Vergin et al., 2013b). Our study extends the historical dataset, described above, to finer taxonomic scales (Supplementary Table 3) and links bacterial community data to corresponding dynamics of DOM, TDAA, and dissolved metabolites dynamics.

## Conclusion

Complementary measurements of DOM biogeochemistry and microbial communities revealed a more detailed picture of the changing diagenetic status of DOM in the context of physical water displacements associated with seasonal mixing. TDAA and dissolved metabolite indices provided insight into complex temporal patterns of DOM release, uptake, and potential interactions with multiple biological processes in seawater. Studying temporal and spatial variability of DOM and bacterial communities associated with deep convective mixing will lead to a more detailed understanding of the vertical carbon flux and microbial processes that alter the quantity and quality of DOM in the ocean.

## Data Availability Statement

BIOS-SCOPE cruise data presented in this study are deposited in the BCO-DMO repository, Available at: http://lod.bco-dmo.org/id/dataset/861266. BATS cruise data presented in this study are deposited in the BCO-DMO repository, Available at: http://lod.bco-dmo.org/id/dataset/3782, and BATS data repository, Available at: http://bats.bios.edu/bats-data/. 16S amplicon sequences presented in this study are deposited in the NCBI SRA repository, project number PRJNA769790. Targeted metabolomics data presented in this study are deposited at MetaboLights repository, accession number MTBLS2356.

## Author Contributions

SL and CC conceived the data integration and wrote the manuscript. SL, KL, EK, KV, LB, SG, RP, KO, EH, DH, RJ, and RC collected and analyzed the samples. SL, KL, KV, LB, RP, KO, EH, DH, RJ, and RC analyzed the data. All authors reviewed and contributed to the manuscript writing.

## Conflict of Interest

KV was employed by Microbial DNA Analytics. The remaining authors declare that the research was conducted in the absence of any commercial or financial relationships that could be construed as a potential conflict of interest.

## Publisher’s Note

All claims expressed in this article are solely those of the authors and do not necessarily represent those of their affiliated organizations, or those of the publisher, the editors and the reviewers. Any product that may be evaluated in this article, or claim that may be made by its manufacturer, is not guaranteed or endorsed by the publisher.

## Footnotes

1
https://www.ebi.ac.uk/metabolights/MTBLS2356

2
http://lod.bco-dmo.org/id/dataset/3782

3
http://bats.bios.edu/bats-data/
