# Supplementary material for: Linkages Among Dissolved Organic Matter Export, Dissolved Metabolites, and Associated Microbial Community Structure Response in the Northwestern Sargasso Sea on a Seasonal Scale
Source: Front Microbiol. 2022 Mar 8;13:833252. doi: 10.3389/fmicb.2022.833252 (PMC8957919; doi:10.3389/fmicb.2022.833252)
Supplement: Supplementary file 1 [file Data_Sheet_1.PDF]

Table S1. Mol% of each amino acid in all samples. Asp: aspartic acid; Glu: glutamic acid; His: histidine; Ser: serine; Arg: arginine; Thr: threonine; Gly: glycine; BAla: β-alanine; Tyr: tyrosine; Ala: alanine; GABA: γ-aminobutyric acid; Met: methionine; Val: valine; Ile: isoleucine; Leu: leucine; Lys: lysine.

Time	Cruise	Depth (m)	Asp	Glu	His	Ser	Arg	Thr	Gly	BAla	Tyr	Ala	GABA	Met	Val	Ile	Leu	Lys
July 2016	BIOS-SCOPE AE1614	1	11.41	5.85	0.00	12.01	3.15	5.14	32.89	4.68	0.70	17.74	0.84	0.14	2.79	0.22	0.88	1.57
		40	12.33	7.36	0.33	7.88	3.37	7.36	32.25	4.70	0.46	16.45	0.88	0.24	3.18	0.07	0.83	2.31
		120	12.78	7.35	0.00	8.57	3.45	6.16	31.85	4.10	0.33	16.65	0.60	0.26	3.90	0.41	1.05	2.54
		200	11.39	6.12	0.00	7.06	3.69	5.38	37.88	5.24	0.61	16.73	0.50	0.01	2.73	0.00	0.72	1.96
		300	14.13	8.33	0.00	8.26	3.42	4.53	34.19	4.39	0.00	16.99	0.29	0.00	3.26	0.00	0.00	2.22
		1	13.75	11.78	0.00	9.26	3.91	6.19	27.91	4.17	0.62	14.27	1.26	0.32	2.78	0.36	1.49	1.93
		40	12.41	8.00	1.47	14.05	2.08	6.80	30.20	3.45	0.65	14.54	1.26	0.07	2.69	0.29	0.53	1.51
		120	14.10	9.84	0.00	5.01	3.26	6.11	34.32	6.15	0.05	16.31	0.93	0.27	2.57	0.21	0.45	0.44
		200	12.92	7.07	0.00	3.43	9.10	2.33	32.84	4.74	0.31	17.84	5.50	0.17	0.89	0.15	1.13	1.59
		300	13.55	12.36	0.03	4.47	2.42	3.97	37.86	4.52	0.48	13.42	1.61	0.01	3.06	0.00	1.22	1.01
Sept. 2016	BIOS-SCOPE AE1620	1	11.44	11.41	0.00	3.96	2.99	5.42	35.36	5.88	0.34	15.84	1.33	0.34	1.89	2.16	0.57	1.07
		40	14.48	10.15	0.00	2.42	2.65	4.99	35.86	5.69	0.56	15.22	0.63	0.48	2.91	1.61	0.26	2.10
		120	14.52	15.51	0.00	2.18	2.71	4.05	29.89	4.81	0.00	14.30	1.28	0.20	3.53	2.23	3.45	1.34
		200	9.52	7.94	0.00	0.00	0.50	1.06	54.14	7.48	0.00	16.59	1.82	0.30	0.20	0.46	0.00	0.01
		300	14.98	4.73	0.00	0.00	0.00	0.39	45.19	10.60	0.00	18.09	5.49	0.47	0.00	0.07	0.00	0.00
		1	11.22	6.10	0.00	9.81	3.69	6.00	32.46	4.59	0.49	16.79	1.42	0.49	2.95	1.07	1.60	1.31
		120	10.97	6.10	0.00	9.94	2.57	6.00	34.02	4.72	0.32	17.24	1.91	0.54	2.44	1.02	1.16	1.06
		200	10.54	6.97	0.27	11.37	3.16	5.02	33.04	3.82	0.78	16.10	1.95	0.70	2.49	1.48	1.10	1.20
		300	11.23	10.09	0.00	8.75	3.56	5.50	28.64	3.45	1.23	13.92	1.24	0.67	3.23	2.19	4.15	2.13
Oct. 2016	BATS 10329	1	11.87	10.21	0.00	11.02	4.35	7.32	25.78	3.64	1.12	15.36	0.92	0.00	3.18	0.34	1.69	3.19
		40	11.61	9.07	0.00	10.33	3.79	5.24	29.24	4.00	1.13	17.40	0.69	0.00	3.19	0.67	1.61	2.04
		120	11.36	8.45	0.87	4.36	2.68	5.94	35.06	5.11	0.32	19.53	0.46	0.00	2.73	0.68	0.32	2.14
		200	10.62	3.00	0.00	1.51	1.59	4.40	45.96	7.48	0.61	20.59	0.30	0.00	1.26	0.16	0.91	1.61
		300	10.80	6.89	0.00	8.80	2.69	6.91	35.44	5.19	1.05	20.67	0.51	0.00	0.97	0.00	0.00	0.10
Dec. 2016	BATS 10331	1	17.60	9.85	0.24	7.24	2.26	4.83	29.92	4.21	0.76	17.00	0.16	0.98	2.44	0.55	0.86	1.10
		40	16.34	11.11	0.13	6.94	2.48	6.95	27.28	3.45	1.13	16.12	0.08	0.69	2.69	0.80	1.91	1.89
		120	16.67	9.92	0.10	4.91	1.25	5.50	32.68	3.67	0.43	19.93	0.14	0.91	2.93	0.28	0.00	0.68
		200	18.53	10.62	0.37	5.41	1.66	3.92	32.65	3.70	0.38	16.91	0.00	0.93	2.70	0.62	0.19	1.41
		300	15.71	11.43	0.79	7.18	2.03	3.36	33.08	2.33	1.49	14.73	0.00	1.05	2.49	0.37	1.76	2.19
Feb. 2017	BATS 10333	1	14.81	8.74	0.00	9.20	3.82	3.25	30.22	5.02	0.97	14.76	1.99	0.00	2.86	0.57	1.47	2.34
		40	12.02	6.60	0.00	7.23	4.71	4.37	35.66	6.02	0.63	14.01	1.70	0.09	1.78	0.32	1.29	3.57
		120	12.69	9.67	1.39	4.52	5.91	4.89	31.84	6.89	0.85	13.24	2.19	0.79	1.56	0.16	0.92	2.48
		200	15.70	6.00	0.00	3.45	3.01	3.18	38.82	6.71	0.21	16.20	0.71	0.19	2.40	0.34	1.10	1.98
		300	15.21	7.19	0.00	3.34	2.58	1.74	35.51	7.55	1.11	14.75	5.32	0.37	2.29	0.19	0.27	2.59

Tabls S1. continued

Apr. 2017	BIOS-SCOPE 1703	1	13.40	7.60	0.00	2.78	2.40	6.42	36.71	4.52	0.00	19.19	1.37	0.03	2.55	0.46	0.24	2.33
		40	12.79	10.34	0.00	5.33	2.08	5.75	34.15	3.04	0.59	15.86	1.29	0.05	3.56	1.63	0.74	2.81
		120	12.48	8.07	0.00	6.60	1.68	5.86	34.75	3.52	0.16	17.09	2.33	0.00	3.02	1.24	0.72	2.47
		200	11.94	7.17	0.00	4.20	1.06	6.45	37.72	3.61	0.21	19.96	1.16	0.15	2.54	1.62	0.35	1.86
		300	13.91	7.80	0.00	2.88	1.01	5.86	37.55	3.49	0.36	17.64	1.12	0.13	3.33	1.67	0.97	2.29
		1	11.07	8.17	0.00	8.12	3.33	4.54	35.81	3.07	1.00	14.83	0.49	0.32	3.30	1.34	2.63	1.97
		40	10.11	7.78	0.00	9.34	3.24	6.56	31.99	2.60	0.91	17.41	0.56	0.32	3.86	1.26	1.74	2.31
		120	11.18	8.45	0.00	9.46	3.49	6.51	29.61	2.42	1.03	16.36	0.59	0.39	4.35	1.77	1.97	2.42
		200	12.60	7.86	0.00	8.97	3.73	6.37	31.18	2.92	0.69	16.67	1.65	0.33	3.27	1.21	0.90	1.66
		300	11.74	8.68	0.40	10.23	3.38	4.79	28.92	2.72	1.64	13.72	2.46	0.17	4.09	2.05	2.74	2.27
May 2017	BATS 10335	1	12.90	8.34	0.00	6.73	4.56	8.50	28.70	5.55	1.11	17.20	0.00	0.12	3.13	0.81	0.84	1.50
		40	15.32	12.21	0.21	7.83	4.62	6.96	24.73	4.18	1.43	15.84	0.00	0.17	3.47	1.18	0.44	1.43
		120	13.94	12.53	0.00	8.23	4.07	5.11	29.64	4.42	1.60	15.34	0.00	0.03	2.62	1.04	0.00	1.44
		200	17.63	14.77	0.01	8.10	3.79	5.35	24.55	3.47	1.68	14.79	0.00	0.09	3.16	1.07	0.00	1.54
		300	13.21	10.89	0.56	11.99	4.57	6.32	27.79	3.12	1.54	12.98	0.00	0.03	3.18	0.92	0.93	1.98
June 2017	BATS 10336	1	13.88	6.25	0.00	4.95	2.61	6.34	33.53	6.24	0.14	18.22	2.11	1.26	2.11	0.00	0.85	1.50
		40	16.08	8.81	0.00	4.66	3.11	4.35	30.76	4.84	0.09	16.41	2.79	1.05	2.66	0.52	2.02	1.84
		120	15.04	9.34	0.06	3.86	2.44	5.31	30.25	4.92	0.13	16.70	3.48	2.09	2.38	0.25	1.55	2.19
		200	16.79	8.86	0.00	4.20	2.31	5.02	31.55	5.18	0.13	17.79	2.78	1.73	2.18	0.00	0.07	1.41
		300	23.57	12.04	0.00	4.63	1.32	4.35	25.63	4.10	0.00	17.18	1.88	0.43	3.33	0.48	0.00	1.06
July 2017	BIOS-SCOPE 1712	1	10.76	9.34	0.00	9.19	4.63	1.68	29.22	3.89	1.34	15.49	0.95	0.48	4.57	1.62	3.15	3.69
		40	8.98	9.32	0.03	10.60	4.08	2.78	32.33	4.59	1.25	15.66	0.53	0.14	3.64	1.14	2.05	2.88
		120	10.66	10.40	0.00	9.70	3.32	1.13	33.40	5.23	1.11	15.96	0.38	0.00	2.77	0.45	0.36	5.14
		200	9.65	9.70	0.00	12.20	4.58	0.27	33.41	3.78	1.32	14.71	1.17	0.19	2.94	0.97	1.88	3.22
		300	9.81	7.76	0.00	12.37	3.19	0.02	34.95	4.78	1.09	16.77	1.81	0.52	2.86	0.48	0.48	3.09
		1	13.59	11.24	0.00	3.87	3.07	4.42	31.65	4.90	0.70	17.55	1.18	0.16	3.75	0.94	1.22	1.75
		40	10.25	12.83	0.00	5.45	2.53	3.22	34.81	4.51	0.77	17.94	0.78	0.00	3.60	0.50	0.97	1.84
		120	13.26	11.07	0.00	0.26	2.07	2.63	38.34	6.14	0.21	19.31	0.51	0.00	3.08	0.69	0.38	2.04
		200	12.22	8.62	0.00	3.71	2.31	3.01	38.81	6.03	0.32	20.86	0.45	0.00	1.68	0.67	0.37	0.92
		300	13.84	11.13	0.00	0.35	1.69	1.94	40.04	6.44	0.16	19.61	0.00	0.00	2.41	0.88	0.45	1.06
Aug. 2017	BATS 10338	1	13.48	8.59	0.00	7.80	4.96	5.77	25.59	4.89	0.29	18.65	1.13	0.92	2.93	1.02	1.94	2.03
		40	11.62	6.10	0.00	8.00	4.12	7.91	28.20	4.65	0.66	17.13	1.13	1.16	2.80	1.30	2.62	2.60
		120	14.07	9.70	0.80	8.74	3.44	4.21	28.45	3.92	0.90	16.32	0.72	0.63	2.80	1.43	1.62	2.26
		200	17.36	7.79	0.14	8.03	2.70	6.51	27.82	4.49	0.21	17.69	0.64	1.00	2.63	1.08	0.45	1.47
		300	15.12	5.96	0.00	9.58	2.82	6.06	28.89	4.43	0.33	18.55	0.89	1.36	2.97	1.43	0.05	1.54
