# Supplementary material for: Linkages Among Dissolved Organic Matter Export, Dissolved Metabolites, and Associated Microbial Community Structure Response in the Northwestern Sargasso Sea on a Seasonal Scale
Source: Front Microbiol. 2022 Mar 8;13:833252. doi: 10.3389/fmicb.2022.833252 (PMC8957919; doi:10.3389/fmicb.2022.833252)
Supplement: Supplementary file 2 [file Data_Sheet_2.PDF]

Table S2. Summary of categories of metabolites quantified in the present project. One category for each metabolite is shown in Figure 3, a more complete set of options is provided in this table. The extraction efficiency information is from Johnson et al. (2017) using Vinyard Sound Water matrix (seawater matrix).

**compound**	**Extraction %**	**KEGG**	**category**	**alternate categories**
2,3-dihydroxybenzoic acid	100.9	C00196	benzoic acids	
2,3-dihydroxypropane-1-sulfonate	0.6	C19675	Dissolved organic sulfur	
3-mercaptopropionic acid	88.6		Dissolved organic sulfur	
3-methyl-2-oxobutanoic acid	10.1	C00141	Amino acid precursors	
4-aminobenzoic acid	18.5	C00568	benzoic acids	Dissolved organic nitrogen
4-hydroxybenzoic acid	88	C00156	benzoic acids	
5-methylthioadenosine	80.9	C00170	Dissolved organic sulfur	Dissolved organic nitrogen
adenosine	6.5	C00212	Nucleic acid precursors	Dissolved organic nitrogen
biotin	52.8	C00120	vitamins	Dissolved organic sulfur, dissolved organic nitrogen
caffeine	23.5	C07481	Dissolved organic nitrogen	
chitotriose	5.6	n/a	Dissolved organic nitrogen	
cyanocobalamin	79.1	C08230	vitamins	Dissolved organic phosphorus, dissolved organic nitrogen
D-Ribose 5-phosphate	0.1	C00117	Dissolved organic phosphorus	
desthiobiotin	6.5	C01909	vitamins	Dissolved organic nitrogen
folic acid	41.2	C00504	vitamins	Dissolved organic nitrogen
glutathione	1.1	C00051	Dissolved organic sulfur	Dissolved organic nitrogen
glutathione oxidized	1.5	C00127	Dissolved organic sulfur	Dissolved organic nitrogen
glyphosate	15.1	C01705	Dissolved organic phosphorus	Dissolved organic nitrogen
guanosine	7.7	C00387	Nucleic acid precursors	Dissolved organic nitrogen
indole 3-acetic acid	16.8	C00954	Dissolved organic nitrogen	
inosine	8.1	C00294	Nucleic acid precursors	Dissolved organic nitrogen
isoleucine	1.41	C00407	amino acids	Dissolved organic nitrogen
kynurenine	41.7	C00328	Dissolved organic nitrogen	
leucine	3.3	C00123	amino acids	Dissolved organic nitrogen
malic acid	0.7	C00149	other	
n-acetyl glutamic acid	1.1	C00624	Dissolved organic nitrogen	
n-acetyl muramic acid	2.8	C02713	Dissolved organic nitrogen	
nicotinamide adenine dinucleotide	20.9	C00004	Dissolved organic phosphorus	Dissolved organic nitrogen
pantothenic acid	51.9	C00864	vitamins	Dissolved organic nitrogen
phenylalanine	39.7	C00079	amino acids	Dissolved organic nitrogen
phosphoglyceric acid	1.7		Dissolved organic phosphorus	Dissolved organic nitrogen
pyridoxine	6.8	C00314	vitamins	Dissolved organic nitrogen
riboflavin	87.6	C00255	vitamins	Dissolved organic nitrogen
S-(5-adenosyl)-L-homocysteine	43.3	C00021	Dissolved organic sulfur	Dissolved organic nitrogen
syringic acid	32.9	C10833	other	
taurocholic acid	92.8	C05122	Dissolved organic sulfur	Dissolved organic nitrogen
thymidine	52.6	C00214	Nucleic acid precursors	Dissolved organic nitrogen
tryptamine	21.3	C00398	Dissolved organic nitrogen	
tryptophan	46.7	C00078	amino acids	Dissolved organic nitrogen
tyrosine	2.1	C00082	amino acids	
xanthosine	10.4	C01762	Nucleic acid precursors	Dissolved organic nitrogen
