# Supplementary material for: Linkages Among Dissolved Organic Matter Export, Dissolved Metabolites, and Associated Microbial Community Structure Response in the Northwestern Sargasso Sea on a Seasonal Scale
Source: Front Microbiol. 2022 Mar 8;13:833252. doi: 10.3389/fmicb.2022.833252 (PMC8957919; doi:10.3389/fmicb.2022.833252)
Supplement: Supplementary file 3 [file Data_Sheet_3.PDF]

Table S3**.** Summary of ASVs (name grouped by phylum or class name (bold)) within the top 300 ASVs showing enrichment in relative contribution during or shortly following convective mixing in the mesopelagic. Only ASVs with a significant positive correlation to DOC or TDAA C are shown, and the table presents the lagged month of ASV 120-300 m mean relative abundance relative to DOC and/or TDAA C (zero means no lag, negative values mean changes of 120-300 m mean ASV relative abundance lagged the change of 120-300 m mean DOC and/or TDAA C) and max positive CCF correlation coefficient. Only negative lags were considered as those are the cases where DOC or TDAA lead changes in the ASVs.

		**significantly cross-correlated with DOC**		**significantly cross-correlated with TDAA C**	
**Name**	**ASV ID**	**Lag**	**CCF**	**Lag**	**CCF**
Alphaproteobacteria					
SAR11_clade_Ia.3	ASV8	-4	0.63	0	0.66
SAR11_clade_Ia.3	ASV74	-4	0.59	-2	0.66
SAR11_clade_Ia.3	ASV14485	-5	0.52	-1	0.65
SAR11_clade_Ia.3	ASV14512	-6	0.32	-1	0.56
SAR11_clade_Ia.3	ASV15			0	0.76
SAR11_clade_Ia.3	ASV42			0	0.68
SAR11_clade_Ia.3	ASV43			0	0.64
SAR11_clade_Ia.3	ASV76			0	0.73
SAR11_clade_Ia.3	ASV14498			-1	0.67
SAR11_clade_Ia.3	ASV14511			-1	0.63
SAR11_clade_Ia.4	ASV143	-2	0.62	0	0.57
SAR11_clade_Ia.4	ASV40			0	0.66
SAR11_clade_Ia.4	ASV14482			-2	0.51
SAR11_clade_Ia.4	ASV14483			-2	0.59
SAR11_clade_Ib.1	ASV7	-4	0.59	0	0.72
SAR11_clade_Ib.2	ASV5	-2	0.56	0	0.66
SAR11_clade_Ib.2	ASV22	-2	0.60		
SAR11_clade_Ib.2	ASV140	-2	0.60		
SAR11_clade_Ib.2	ASV14494	-3	0.54	-1	0.73
SAR11_clade_Ib.2	ASV243			0	0.67
SAR11_clade_Id	ASV190	-2	0.65		
SAR11_clade_Id	ASV14508			-1	0.53
SAR11_clade_IIa.A	ASV156	-2	0.54		

Table S3. continued.

Name	ASV ID	significantly cross-correlated with DOC		significantly cross-correlated with TDAA C	
		Lag	CCF	Lag	CCF
SAR11_clade_IIa.A	ASV36			0	0.72
SAR11_clade_IIa.A	ASV164			0	0.66
SAR11_clade_IIa.B	ASV232	-1	0.57		
SAR11_clade_IIa.B	ASV25			0	0.63
SAR11_clade_IIa.B	ASV14502			-1	0.51
SAR11_clade_IIa	ASV104	-2	0.53	0	0.60
SAR11_clade_IIa	ASV69			0	0.71
SAR11_clade_IIa	ASV14487			-3	0.58
SAR11_clade_IIIa	ASV152			0	0.58
SAR11_clade_IV	ASV95	-1	0.66		
SAR11_clade_IV	ASV108	-1	0.56		
SAR11_clade_IV	ASV92	-2	0.66		
SAR11_clade_IV	ASV262			0	0.58
SAR11_clade	ASV161			0	0.84
OCS116_clade	ASV11	-2	0.67	0	0.54
OCS116_clade	ASV126	-2	0.67		
Rhodobacterales_Rhodobacteraceae	ASV167	-3	0.58		
Rhodospirillales_Rhodospirillaceae_OM75_clade	ASV100	-4	0.68	0	0.68
Rhodospirillales_Rhodospirillaceae_Defluviicoccus	ASV109	-2	0.52	0	0.56
Rhodospirillales_Rhodospirillaceae_AEGEAN.169_marine_group	ASV146	-2	0.61		
Rhodospirillales_Rhodospirillaceae_AEGEAN.169_marine_group	ASV191	-2	0.63		
Rhodospirillales_Rhodospirillaceae_Magnetospira	ASV130			-3	0.58
Rickettsiales_SAR116_clade	ASV123	-4	0.66	0	0.68
Chloroflexi					
SAR202_Clade1	ASV10			0	0.59
SAR202_Clade2	ASV106			-3	0.52
SAR202_Clade2	ASV94			-3	0.56
SAR202_Clade3	ASV119	-6	0.51	-2	0.69
SAR202_Clade	ASV113			0	0.64
Flavobacteriia					
Flavobacteriales_Flavobacteriaceae_NS2b_marine_group	ASV28	-2	0.61		
Flavobacteriales_Flavobacteriaceae_NS2b_marine_group	ASV132	-1	0.52		
Flavobacteriales_Flavobacteriaceae_NS4_marine_group	ASV83	-3	0.54		
Flavobacteriales_Flavobacteriaceae_NS4_marine_group	ASV245	-3	0.56	-1	0.55
Flavobacteriales_Flavobacteriaceae_NS4_marine_group	ASV145	-3	0.52		
Flavobacteriales_Flavobacteriaceae_NS4_marine_group	ASV246	-1	0.62		
Flavobacteriales_Flavobacteriaceae_NS4_marine_group	ASV238	-1	0.59		

Table S3. continued.

Name	ASV ID	significantly cross-correlated with DOC		significantly cross-correlated with TDAA C	
		Lag	CCF	Lag	CCF
Gammaproteobacteria					
Oceanospirillales_SAR86_clade	ASV60	-2	0.60		
Oceanospirillales_SAR86_clade	ASV67	-2	0.55		
Oceanospirillales_SAR86_clade	ASV56	-2	0.66	0	0.59
Oceanospirillales_SAR86_clade	ASV189	-1	0.68		
Oceanospirillales_SAR86_clade	ASV170	-2	0.55		
Oceanospirillales_Oceanospirillaceae_Pseudospirillum	ASV68	-2	0.66		
Oceanospirillales_Oceanospirillaceae_Pseudospirillum	ASV159			-3	0.73
Salinisphaerales_Salinisphaeraceae_ZD0417_marine_group	ASV48	-2	0.62	0	0.68
Salinisphaerales_Salinisphaeraceae_ZD0417_marine_group	ASV111			0	0.73
Alteromonadales_Alteromonadaceae_Alteromonas	ASV11054	-8	0.53	-4	0.52
Alteromonadales_Alteromonadaceae_Alteromonas	ASV2708	-5	0.51	-3	0.67
Cellvibrionales_Halieaceae_OM60.NOR5._clade	ASV198	-3	0.65		
E01.9C.26_marine_group	ASV201			-3	0.59
Verrucomicrobia					
Verrucomicrobiae_Verrucomicrobiales_DEV007	ASV182	-3	0.59		
Acidimicrobiia					
Acidimicrobiales_Sva0996_marine_group	ASV227	-2	0.52		
Acidimicrobiales_Sva0996_marine_group	ASV121			-3	0.65
Acidimicrobiales_Sva0996_marine_group	ASV228			-1	0.60
Acidimicrobiales_OM1_clade_Candidatus_Actinomarina	ASV53	-3	0.57		
Acidimicrobiales_OM1_clade_Candidatus_Actinomarina	ASV18			-1	0.57
Acidimicrobiales_OM1_clade_Candidatus_Actinomarina	ASV58			-1	0.61
Acidimicrobiales_OM1_clade_Candidatus_Actinomarina	ASV230			-1	0.58
Marinimicrobia					
SAR406_clade	ASV79			-3	0.66
SAR406_clade	ASV64			0	0.68
SAR406_clade	ASV97			-3	0.51
SAR406_clade	ASV110			-3	0.51
SAR406_clade	ASV225			-4	0.61
SAR406_clade	ASV147			0	0.64
Deltaproteobacteria					
SAR324_clade_Marine_group_B	ASV45			-3	0.66
Sh765B.TzT.29	ASV148	-2	0.52	0	0.56
