# Supplementary material for: Linkages Among Dissolved Organic Matter Export, Dissolved Metabolites, and Associated Microbial Community Structure Response in the Northwestern Sargasso Sea on a Seasonal Scale
Source: Front Microbiol. 2022 Mar 8;13:833252. doi: 10.3389/fmicb.2022.833252 (PMC8957919; doi:10.3389/fmicb.2022.833252)

Figure S2. Graphical view of how changes in the mixed layer depth alter the concentration of a metabolite due to mixing or changes in metabolite concentration. (A) The metabolite concentrations at a given time (time 0) were integrated to the mixed layer depth at the next time point (t_1 MLD) which results in a value for area t_0. This value was compared to the integrated metabolite value at the next time (time 1), which also integrated to the mixed layer depth at time 1, which results in area t_1. Using the scheme detailed in (A), the x-axis of (B) is the integrated amount of a metabolite in the mixed layer at t_0 (area t_0). The y-axis is the integrated amount of the metabolite at time 1, which has been integrated to the mixed layer depth at t_1 (area t_1). The one-to-one line shows where the concentration of metabolite only changes as a function of mixing. In the figure, for the points away from the one-to-one line, phenylalanine is an example of a metabolite with production > mixing dilution after the April 2017 mixing event compared to 4-hydroxybenzoic acid which showed higher loss compared to dilution after the April 2017 mixing event.

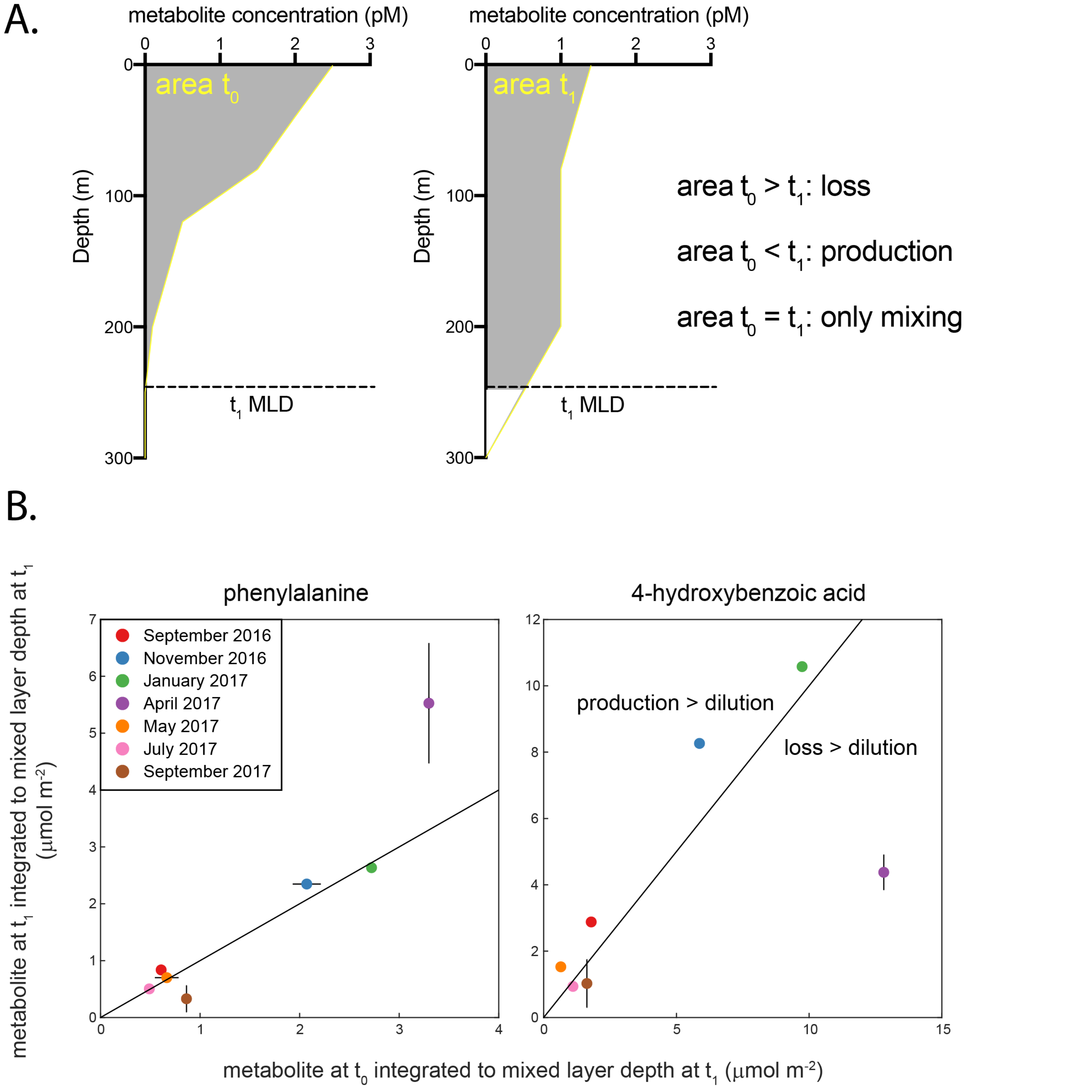

Supplement: Supplementary file 5 [file Data_Sheet_5.PDF]
