# Supplementary material for: Linkages Among Dissolved Organic Matter Export, Dissolved Metabolites, and Associated Microbial Community Structure Response in the Northwestern Sargasso Sea on a Seasonal Scale
Source: Front Microbiol. 2022 Mar 8;13:833252. doi: 10.3389/fmicb.2022.833252 (PMC8957919; doi:10.3389/fmicb.2022.833252)

Figure S3. Graphical view of how changes in the mixed layer depth alter (A) DOC, (B) TDAA-C, and (C) BA due to mixing following similar method shown in Figure S2. The variable at a given time (t0) were integrated to the mixed layer depth (MLD) at the next time point (t1) and compared to the variable value at the next time integrated to the MLD at t1. The one-to-one line shows where the variable only changes as a function of mixing; points above the line indicate production > mixing dilution while points below the line indicate loss > mixing dilution. Asterisk next to legend indicates significant production (**) or loss (*).

(A)

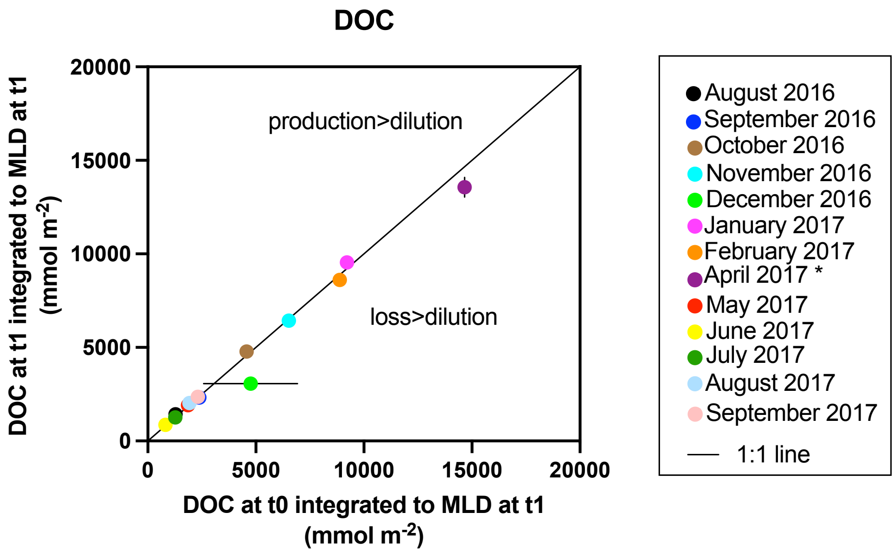

(B)

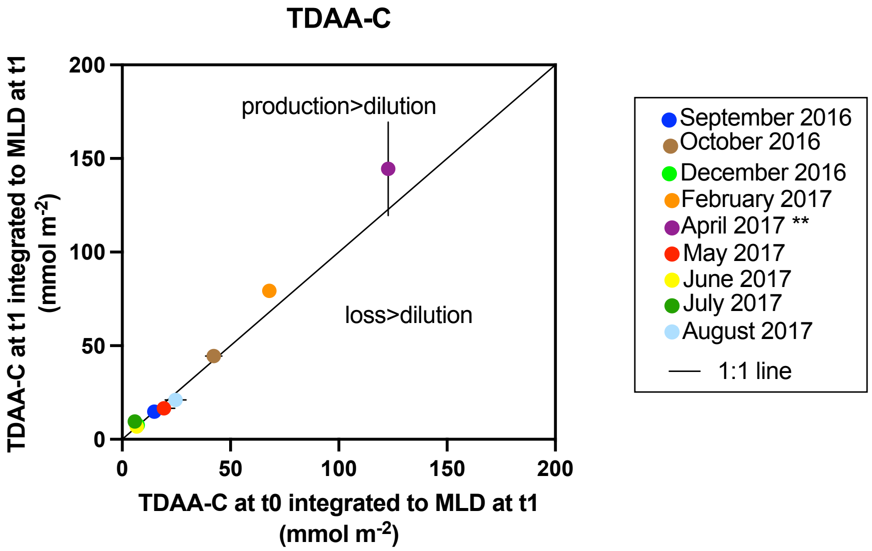

(C)

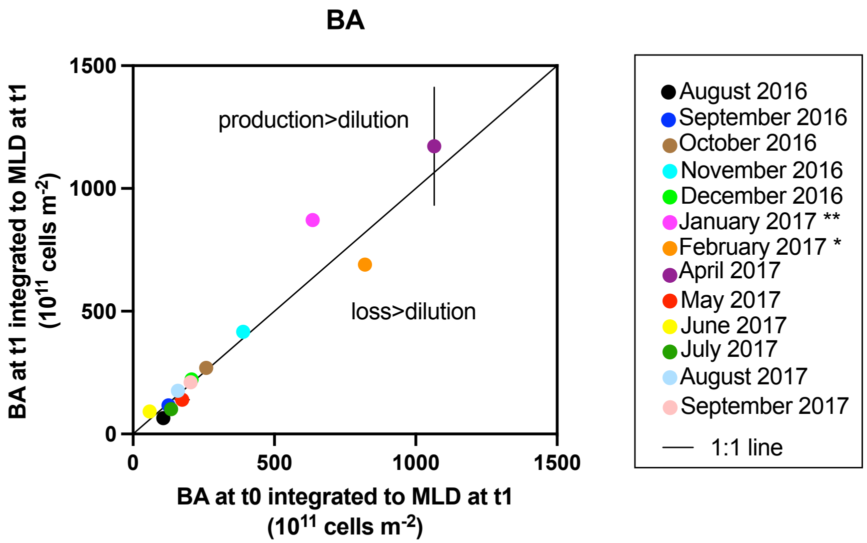

Supplement: Supplementary file 6 [file Data_Sheet_6.PDF]
