# Supplementary material for: Linkages Among Dissolved Organic Matter Export, Dissolved Metabolites, and Associated Microbial Community Structure Response in the Northwestern Sargasso Sea on a Seasonal Scale
Source: Front Microbiol. 2022 Mar 8;13:833252. doi: 10.3389/fmicb.2022.833252 (PMC8957919; doi:10.3389/fmicb.2022.833252)
Supplement: Supplementary file 7 [file Data_Sheet_7.PDF]

Figure S4. Depth profiles of metabolites concentration from July 2016 to Sept 2017, concentrations are given pM with a different color scale for each metabolite. Grey dots indicate sampling depths and black dots indicate the maximum mixed layer depth for the month.

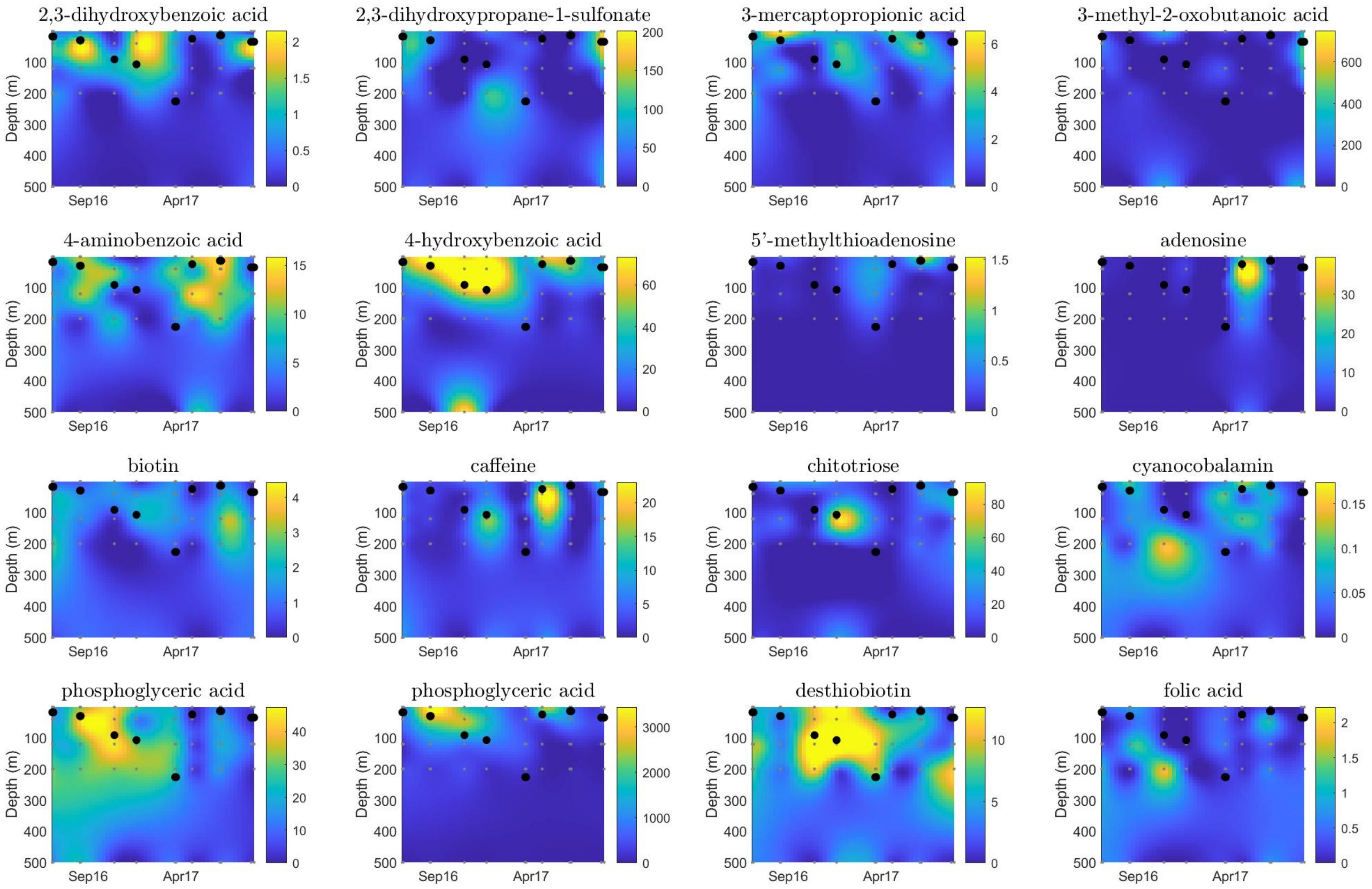

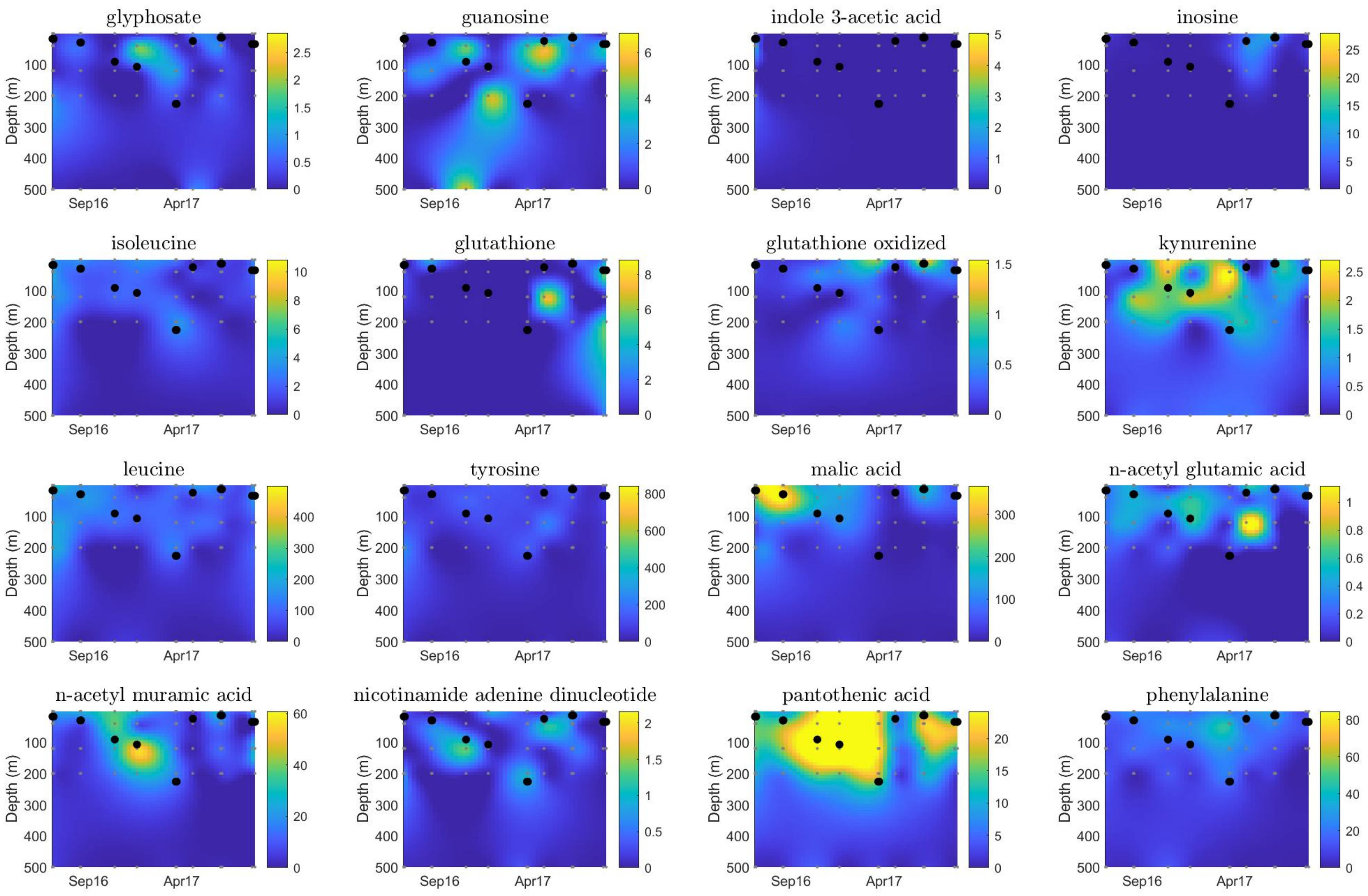
glyphosate
guanosine
indole 3-acetic acid
inosine
isoleucine
glutathione
glutathione oxidized
kynurenine
leucine
tyrosine
malic acid
n-acetyl glutamic acid
n-acetyl muramic acid
nicotinamide adenine dinucleotide
pantothenic acid
phenylalanine
Depth (m)
100
200
300
400
500
Sep16
Apr17

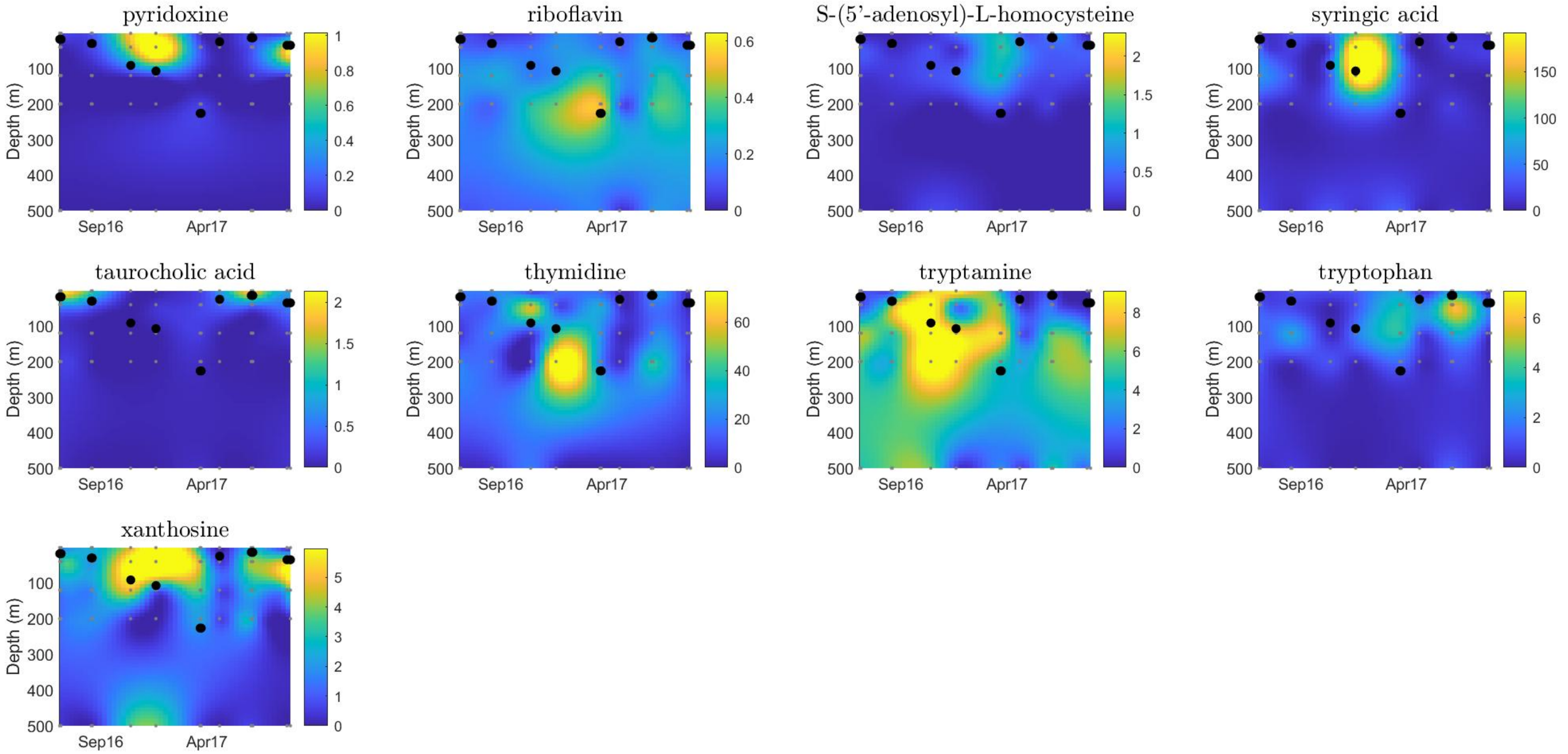
pyridoxine
riboflavin
S-(5'-adenosyl)-L-homocysteine
syringic acid
taurocholic acid
thymidine
tryptamine
tryptophan
xanthosine
Depth (m)
100
200
300
400
500
Sep16
Apr17
