# Supplementary material for: Linkages Among Dissolved Organic Matter Export, Dissolved Metabolites, and Associated Microbial Community Structure Response in the Northwestern Sargasso Sea on a Seasonal Scale
Source: Front Microbiol. 2022 Mar 8;13:833252. doi: 10.3389/fmicb.2022.833252 (PMC8957919; doi:10.3389/fmicb.2022.833252)

Figure S5. Top 300 m depth profiles of relative abundance for ASVs (among top 300 abundant ASVs) that showed enrichment during or shortly after mixing (Apr. 2017-July 2017) in mesopelagic 120-300 m and were significantly cross-correlated with DOC and/or TDAA C. Red dashed rectangle indicates convective mixing time frame and white dashed rectangle indicates 120-300 m mesopelagic depth.

SAR11 clade:

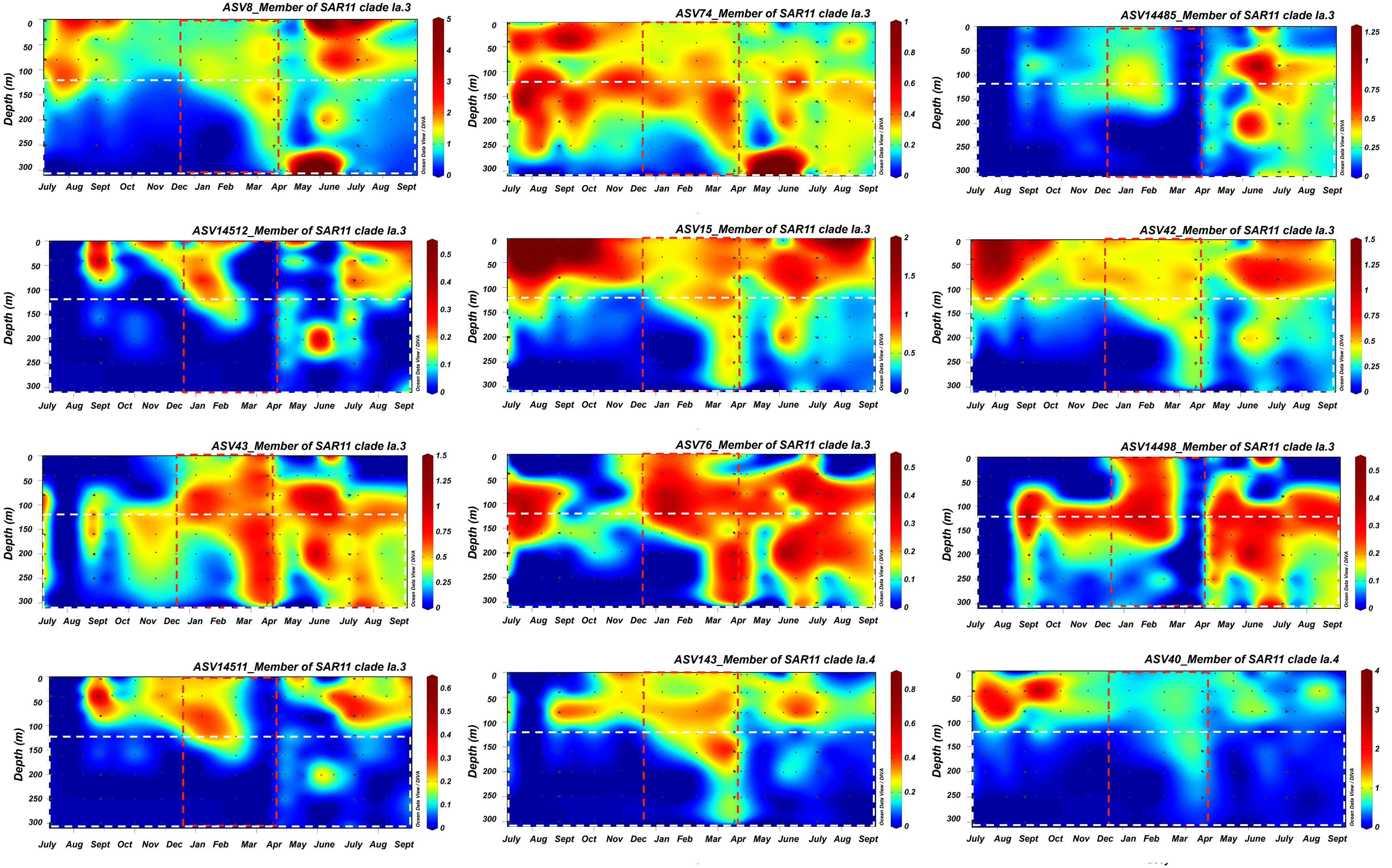

Supplement: Supplementary file 8 [file Data_Sheet_8.PDF]
