# Supplementary figures and images for: Linkages Among Dissolved Organic Matter Export, Dissolved Metabolites, and Associated Microbial Community Structure Response in the Northwestern Sargasso Sea on a Seasonal Scale
Source: Front Microbiol. 2022 Mar 8;13:833252. doi: 10.3389/fmicb.2022.833252 (PMC8957919; doi:10.3389/fmicb.2022.833252)

Figure S5. Continued.

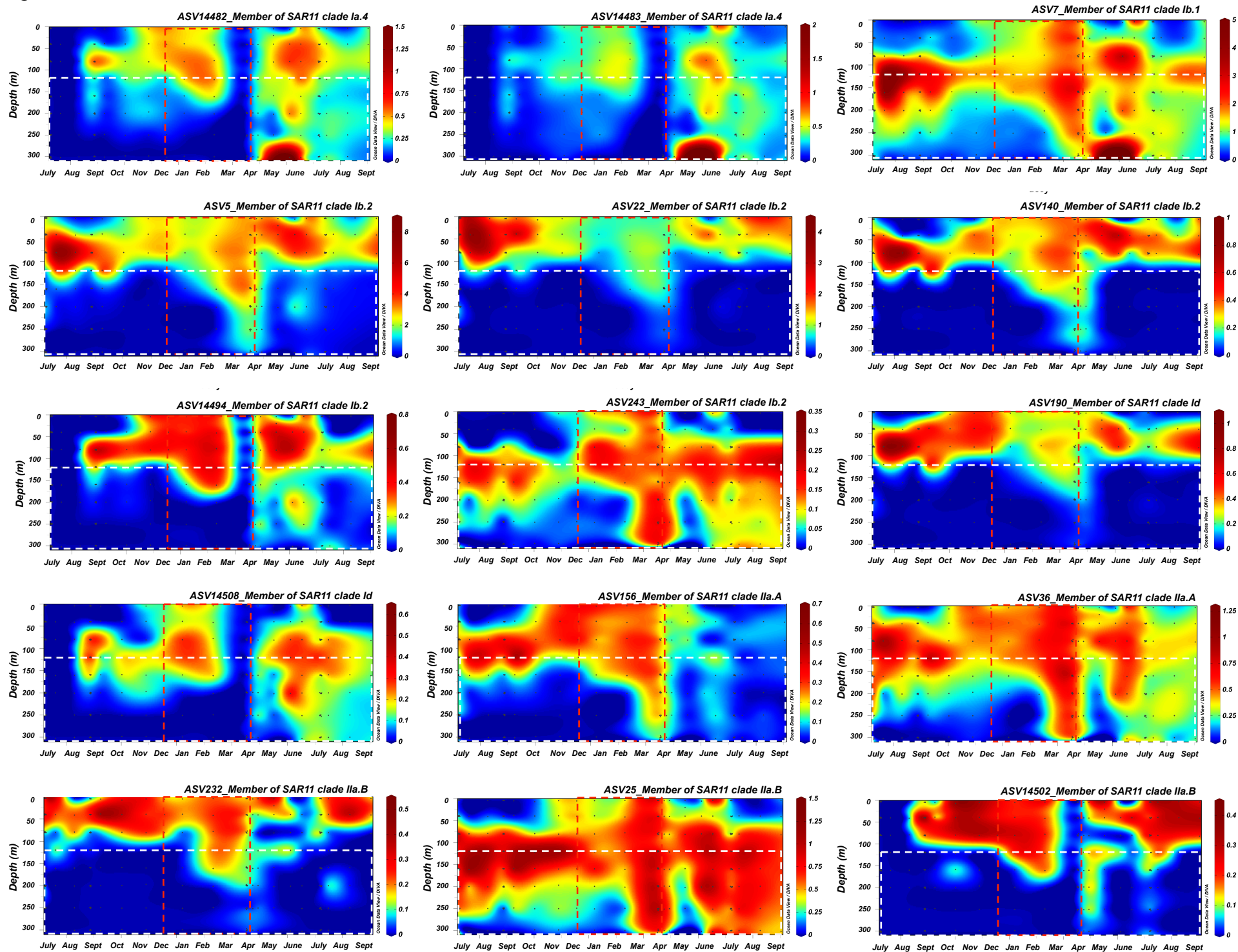

Supplement: Supplementary file 9 [file Data_Sheet_9.PDF]
