# Supplementary material for: Linkages Among Dissolved Organic Matter Export, Dissolved Metabolites, and Associated Microbial Community Structure Response in the Northwestern Sargasso Sea on a Seasonal Scale
Source: Front Microbiol. 2022 Mar 8;13:833252. doi: 10.3389/fmicb.2022.833252 (PMC8957919; doi:10.3389/fmicb.2022.833252)

Figure S5. Continued.

SAR202 clade:

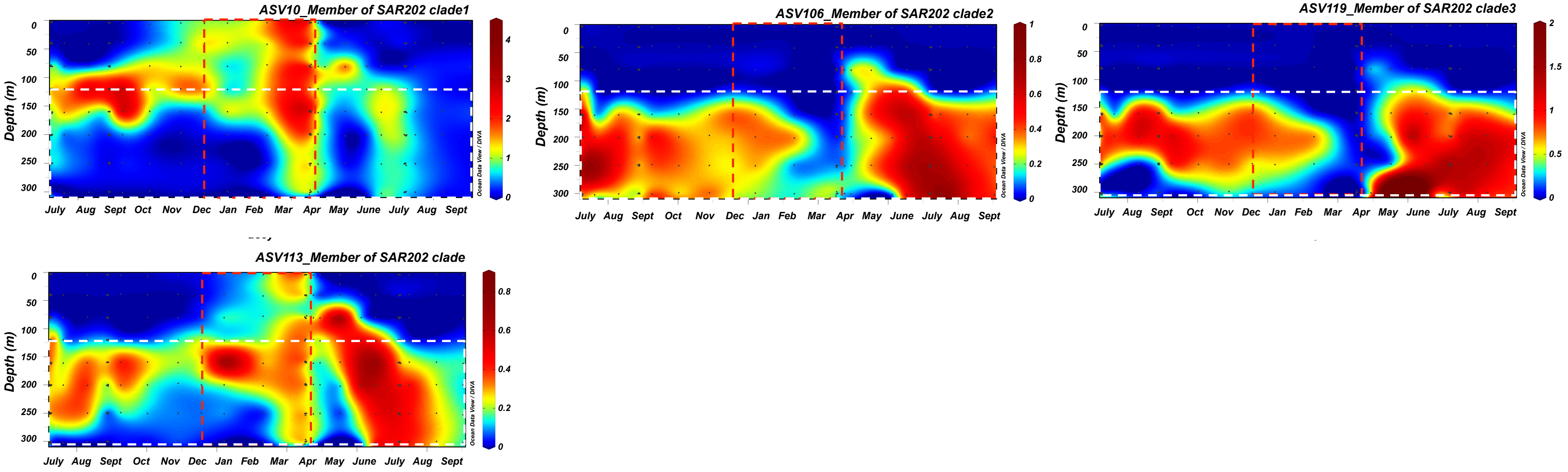

Flavobacterales:

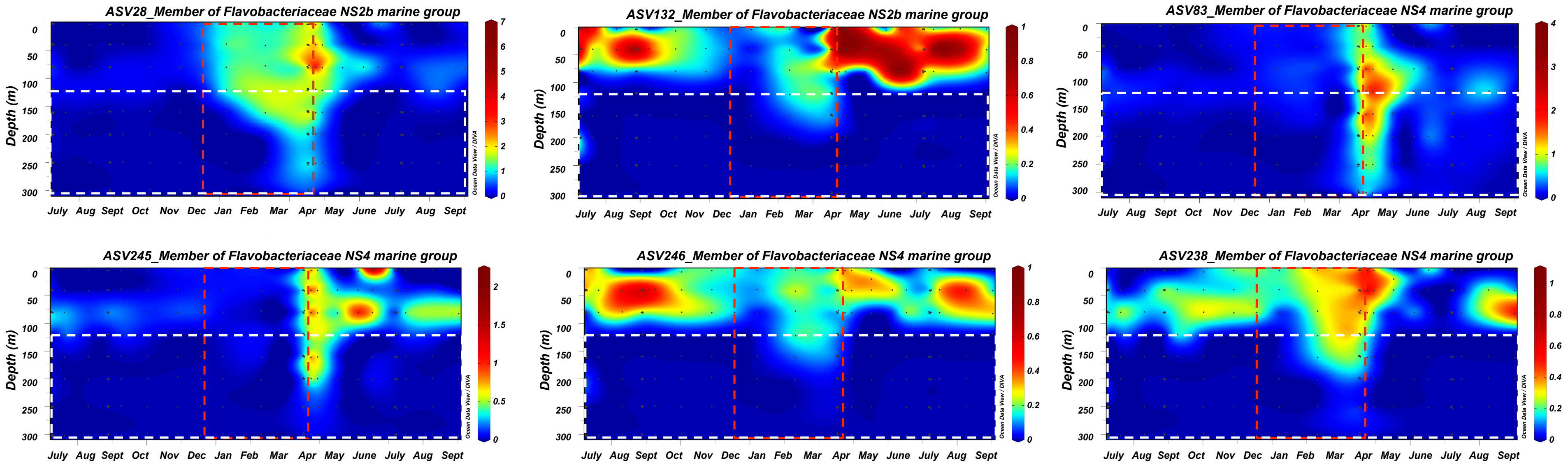

Figure S5. Continued.

Oceanospirillales:

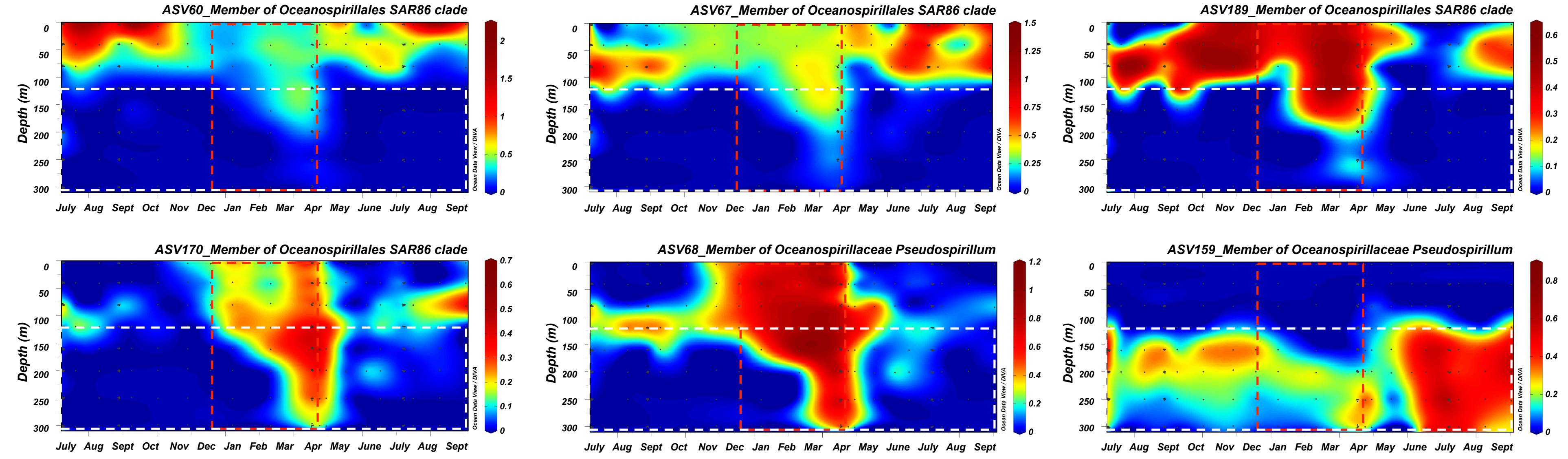

Salinisphaeraceae:

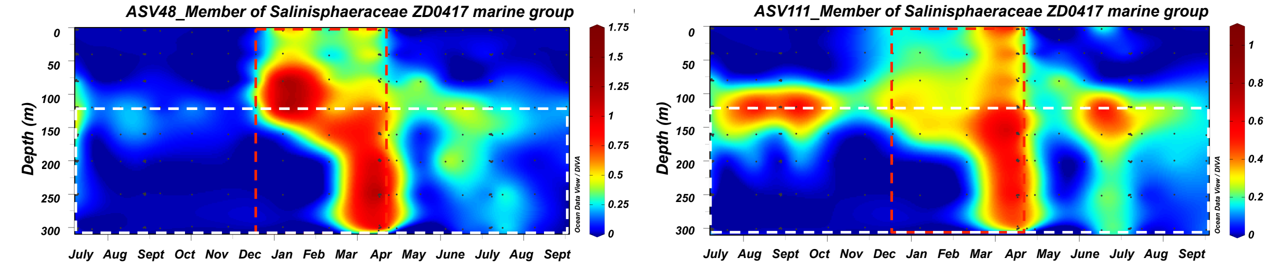

Alteromonas:

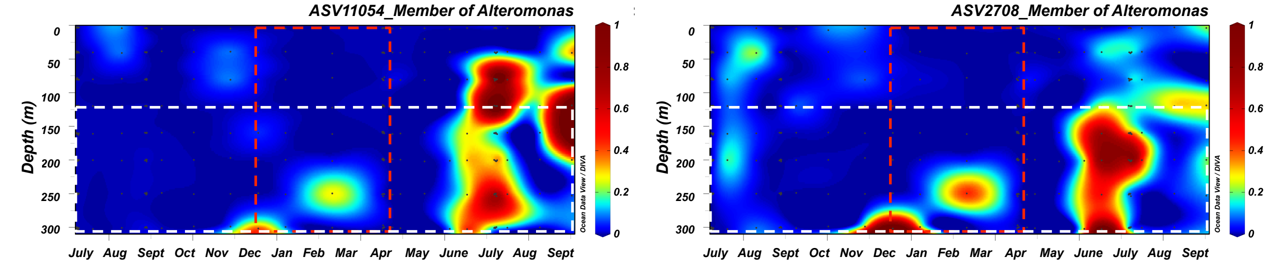

Supplement: Supplementary file 11 [file Data_Sheet_11.PDF]
