# Supplementary material for: Linkages Among Dissolved Organic Matter Export, Dissolved Metabolites, and Associated Microbial Community Structure Response in the Northwestern Sargasso Sea on a Seasonal Scale
Source: Front Microbiol. 2022 Mar 8;13:833252. doi: 10.3389/fmicb.2022.833252 (PMC8957919; doi:10.3389/fmicb.2022.833252)

Figure S5. Continued.

Cellvibrionales:

ASV198_Member of Cellvibrionales Halieaceae OM60 NOR5 clade

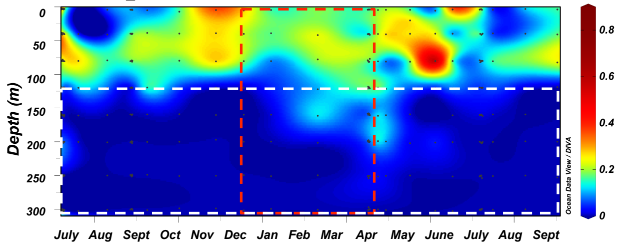

E01.9C.26 marine group:

ASV201_Member of E01.9C.26 marine group

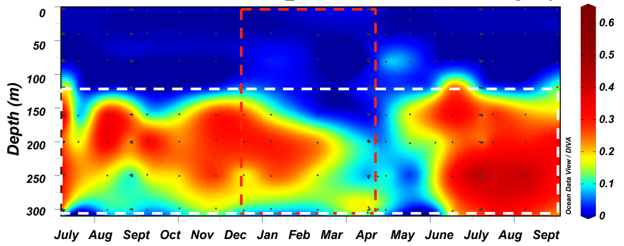

Verrucomicrobiales:

ASV182_Member of Verrucomicrobiales DEV007

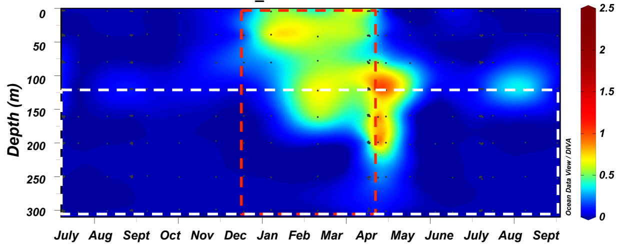

Acidimicrobiales:

ASV227_Member of Acidimicrobiales Sva0996 marine group

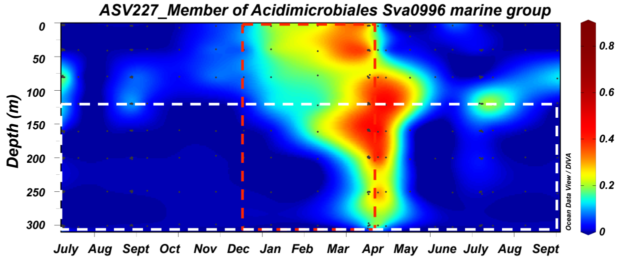

ASV121_Member of Acidimicrobiales Sva0996 marine group

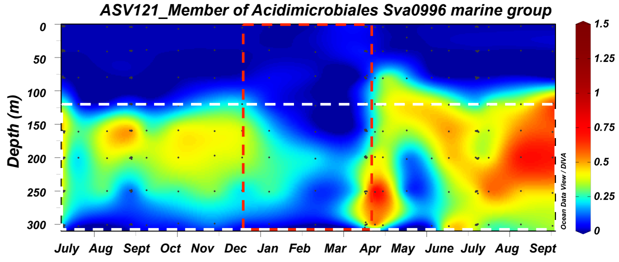

ASV228_Member of Acidimicrobiales Sva0996 marine group

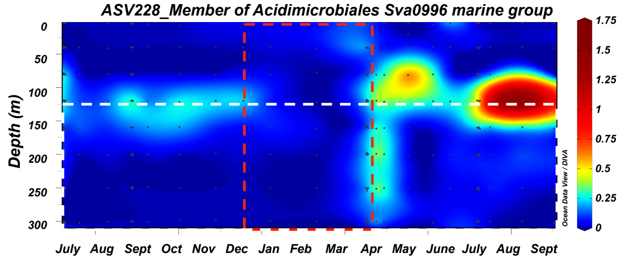

Figure S5. Continued.

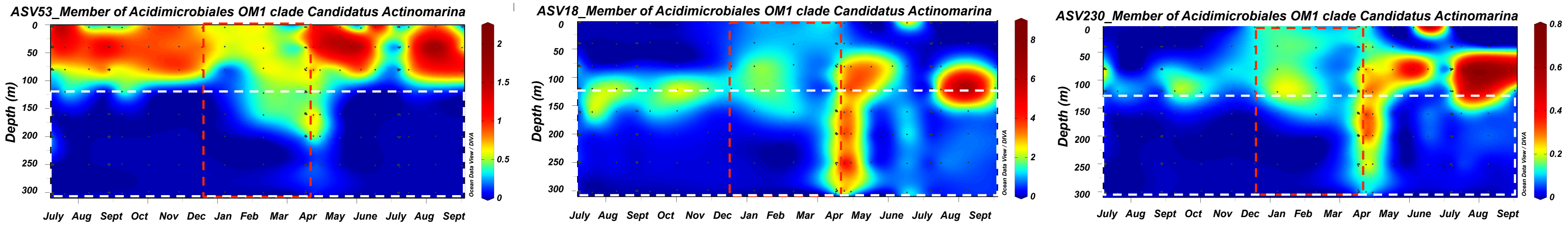

Marinimicrobia:

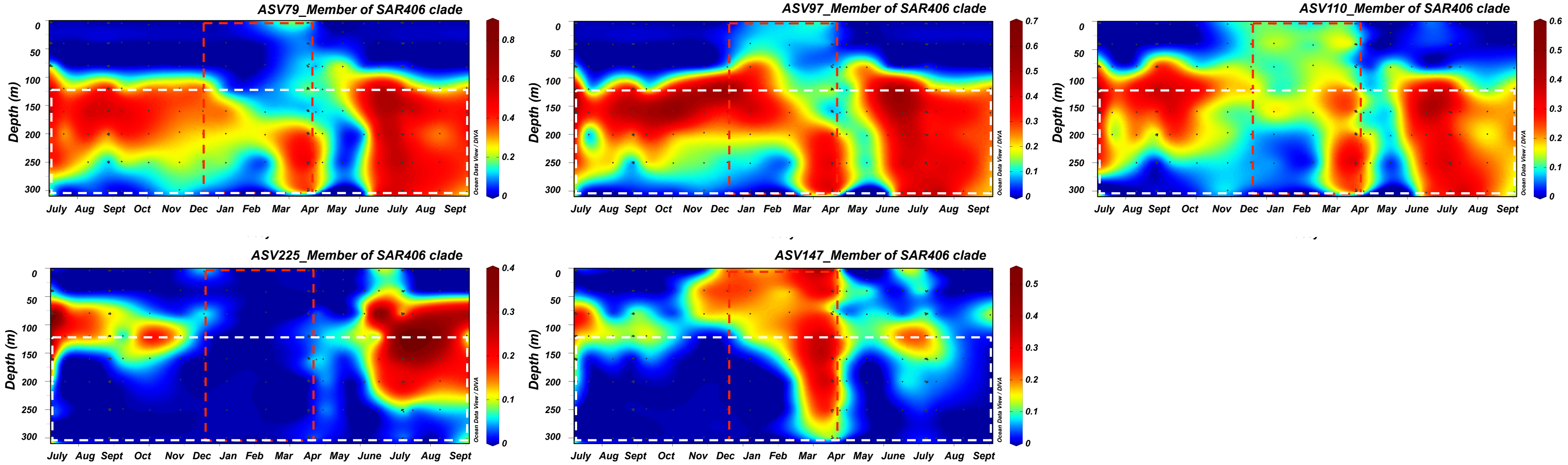

SAR324 clade:

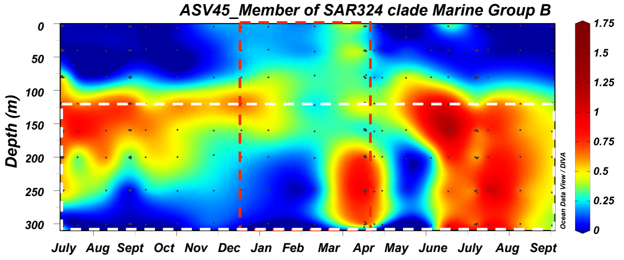

Figure S5. Continued.

Sh765B.TzT.29:

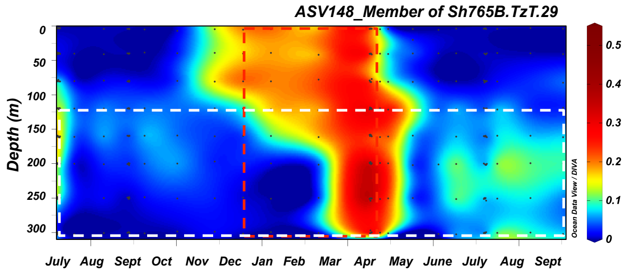

Supplement: Supplementary file 12 [file Data_Sheet_12.PDF]
